# Alternative paths to realize Majorana Fermions in Superconductor-Ferromagnet Heterostructures

**DOI:** 10.1038/s41598-019-42558-3

**Published:** 2019-04-18

**Authors:** G. Livanas, M. Sigrist, G. Varelogiannis

**Affiliations:** 1 Department of Physics, National Technical University of Athens, GR-15780 Athens, Greece; 2 Institut für Theoretische Physik, ETH-Zürich, CH-8093 Zürich, Switzerland

**Keywords:** Superconducting properties and materials, Topological defects

## Abstract

A fundamental obstacle for achieving quantum computation is local decoherence. One way to circumvent this problem rests on the concepts of topological quantum computation using non-local information storage, for example on pairs of Majorana fermions (MFs). The arguably most promising way to generate MFs relies at present on spin-triplet p-wave states of superconductors (SC), which are not abundant in nature, unfortunately. Thus, proposals for their engineering in devices, usually via proximity effect from a conventional SC into materials with strong spin-orbit coupling (SOC), are intensively investigated nowadays. Here we take an alternative path, exploiting the different connections between fields based on a quartet coupling rule for fields introduced by one of us, we demonstrate that, for instance, coexisting Zeeman field with a charge current would provide the conditions to induce p-wave pairing in the presence of singlet superconductivity. This opens new avenues for the engineering of robust MFs in various, not necessarily (quasi-)one-dimensional, superconductor-ferromagnet heterostructures, including such motivated by recent pioneering experiments that report MFs, in particular, without the need of any exotic materials or special structures of intrinsic SOC.

## Introduction

Majorana particles are their own anti-particles^1,2^ each comprising half of a fermion such that widely separated pairs of Majorana states constitute nonlocal fermionic states, immune to local decoherence ideal for building hardware elements for topological quantum computation^3–6^. Spin-triplet p-wave states of superconductors (SC) are known to be potential hosts of MFs although these are rarely intrinsic states of materials^7–9^. In fact, zero-energy Majorana states have been shown on toy models, to emerge at the edges of spinless one-dimensional p-wave SC wires^9^ and in vortex cores of certain two-dimensional chiral *p*_*x*_ + *ip*_*y*_ SC states^8,10,11^.

Given the rarity of convenient p-wave SC in nature, numerous proposals have been put forward for their quantum engineering in devices involving conventional SC instead^12–22^. Especially, quantum engineering procedures of relevant for MF generation effective *p*-wave SC fields from conventional SC in combination with strong SOC materials like Rashba semiconductors^16–18^ or topologic insulators^12,15^, have been implemented with impressive progress^23,24^. In another proposal are not involved strong SOC materials but instead nanowires with special rotating magnetic structures^21^ that are equivalent to nanowires with Rashba SOC.

The most striking and direct experimental evidence of MFs was, however, reported by scanning tunneling microscopy at the edges of ferromagnetic (FM) Fe wires placed on the [110] surface of SC Pb^25^. A convincing explanation of this remarkable phenomenon in terms of a FM atomic chain in proximity with a SC that exhibits strong intrinsic Rashba SOC has been proposed^25–27^. If intrinsic Rashba SOC is so strong on the SC Pb surface then an eventual isolated SC Pb wire with an in-wire field could exhibit at the edges MFs as well, the same could be true at the cores of vortices on eventual SC Pb films.

Here we take an alternative path. Exploring the different connections between the relevant fields based on the *quartet coupling rule* for fields introduced by one of us^28^, we show that appropriate p-wave SC fields and robust MFs may be induced from singlet SC states in the presence of FM and supercurrents, without the need to assume any intrinsic Rashba SOC. Our findings not only provide a groundbreaking perspective on these experiments^25^, they unlock potentially a plethora of related unexplored paths for the quantum engineering of MFs in SC/FM devices in which intelligent combinations of currents and fields play the key role. As a typical example, we propose a versatile trilayer SC/FM/SC device structure that can produce MFs through the same quartets mechanism, illustrating thus how our approach opens new avenues for the controllable quantum engineering of robust MFs in SC/FM heterostructures that may involve trivial materials and may not even need to be quasi-one-dimensional thanks to the directionality of currents.

**Figure 1:**
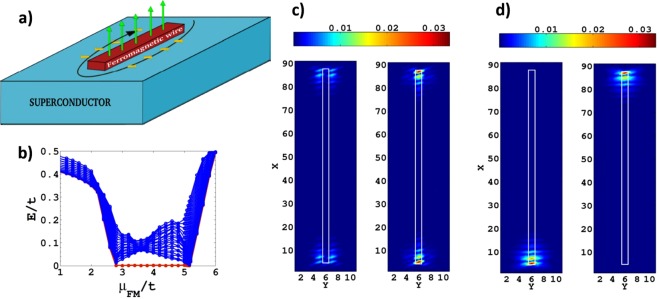
Heterostructure inspired by the experiment^25^. (**a**) One-dimensional FM wire with perpendicular polarization (green arrows) embedded on the surface of a singlet SC, a screening supercurrent in the proximity of the wire flowing around it due to the magnetization of the wire (black arrow) and a small unscreened in plane field component (yellow arrows) in the proximity of the wire. Results remain identical if the sign of the supercurrent and/or the sign of the perpendicular field and/or the sign of all in plane fields is flipped. (**b**) Typical low energy excitation spectrum of Hamiltonian Eq. 1 that contains only the ingredients depicted in (**a**) with Δ = 1 and *μ*_*SC*_ = 0 for the SC region, *h*_*z*_ = 4 in the FM wire, |*h*_*y*_| = 0.4 and |*J*| = 0.2 (all in *t* units), as a function of the chemical potential in the FM wire *μ*_*FM*_. With red line is highlighted the lowest eigenenergy of the system which pins to zero in the non-trivial topological phase emerging approximately for *h*_*z*_−1.2*t* < *μ*_*FM*_ < *h*_*z*_ + 1.2*t*. (**c)** The spin down (left panel) and spin up (right panel) parts of the wave function |Ψ|^2^ corresponding to the zero eigenenergy of the system in the topologically non-trivial phase for *μ*_*FM*_ = *h*_*z*_ = 4. The white rectangle defines the FM wire. (**d)** The same wave function expressed in the Majorana basis (Supplement II) reveals the two Majorana fermions localized respectively on each edge of the FM wire. The white rectangle defines the FM wire.

Inspired by the experiments we start with the presentation of an alternative device setup (see Fig. 1a) to induce *p*-wave superconductivity and MFs using a Zeeman field (FM) and a perimetric supercurrent without relying on intrinsic Rashba SOC. In order to demonstrate the functioning of our design, we introduce here a simple model of a one-dimensional FM nano-wire embedded in the surface of a conventional SC, described by the 2D Hamiltonian ${\mathcal H} ={\sum }_{{\boldsymbol{i}},{\boldsymbol{j}}}\,{{\rm{\Psi }}}_{{\boldsymbol{i}}}^{\dagger }{H}_{{\boldsymbol{i}},{\boldsymbol{j}}}{{\rm{\Psi }}}_{{\boldsymbol{j}}}$ with the necessary and sufficient ingredients depicted in Fig. 1a,

1 $${H}_{i,j}=t{f}_{i,j}{\tau }_{3}+({\mu }_{i}{\tau }_{3}-{\tau }_{3}{{\boldsymbol{h}}}_{i}\cdot \tilde{{\boldsymbol{\sigma }}}+{{\rm{\Delta }}}_{i}{\tau }_{2}{\sigma }_{2}){\delta }_{i,j}+{{\boldsymbol{J}}}_{i}\cdot {{\boldsymbol{g}}}_{i,j},$$

where the Nambu spinor ${{\rm{\Psi }}}_{{\boldsymbol{i}}}^{\dagger }=({\psi }_{{\boldsymbol{i}},\uparrow }^{\dagger },{\psi }_{{\boldsymbol{i}},\downarrow }^{\dagger },{\psi }_{{\boldsymbol{i}},\uparrow },{\psi }_{{\boldsymbol{i}},\downarrow })$ is referring to the electronic states on lattice site ***i***. The Pauli matrices ***τ*** and ***σ*** act on particle-hole and spin space, respectively. The electrons move via nearest-neighbour hopping described by the connection matrix *f*_***i***,***j***_ = *δ*_***j***,***i***±***x***_ + *δ*_***j***,***i***±***y***_ where ***x*** and ***y*** are in-plane unit vectors, with a hopping integral *t*. The local chemical potential is denoted by *μ*_***i***_, ***h***_***i***_ the local vector Zeeman field whereby in our Nambu spinor representation the spin operator is expressed through ${\tau }_{3}\tilde{{\boldsymbol{\sigma }}}={\tau }_{3}({\sigma }_{1},{\tau }_{3}{\sigma }_{2},{\sigma }_{3})$. Moreover, we introduce the pairing field Δ_***i***_ for the conventional SC phase. A further key element is the current ${{\boldsymbol{J}}}_{{\boldsymbol{i}}}=({J}_{{\boldsymbol{i}}}^{x},{J}_{i}^{y})$ with the corresponding connection matrices given by ${{\boldsymbol{g}}}_{{\boldsymbol{i}},{\boldsymbol{j}}}=({g}_{{\boldsymbol{i}},{\boldsymbol{j}}}^{x},{g}_{{\boldsymbol{i}},{\boldsymbol{j}}}^{y})=(\,\pm \,i{\delta }_{{\boldsymbol{j}},{\boldsymbol{i}}\pm {\boldsymbol{x}}},\pm \,i{\delta }_{{\boldsymbol{j}},{\boldsymbol{i}}\pm {\boldsymbol{y}}})$. This is the screening supercurrent due to the magnetization of the ferromagnetic wire.

The setup of our device, as depicted schematically in Fig. 1a, requires that the Zeeman field (magnetic moment) on the FM points along the *z*-axis (perpendicular to the SC surface) and tilts on adjacent sites perpendicular to the wire (*y*-direction). The onsite pairing field Δ_***i***_ is constant over the SC region. The supercurrent flows adjacent to FM wire perimetrically to screen the magnetization of the FM wire. We use a different chemical potential for the FM wire (*μ*_*FM*_) and the SC region (*μ*_*SC*_).

The straightforward numerical calculations of this model yield a quasiparticle (QP) spectrum as presented in Fig. 1b. We observe that a pair of zero energy QP states appear in the range of *μ*_*FM*−_ < *μ*_*FM*_ < *μ*_*FM* +_ with ${\mu }_{FM\mp }\approx {h}_{z}\mp 1.2t$, respectively, for the parameters used (see caption of Fig. 1) and indicate the range in which the FM wire would be metallic in the normal state.

The boundaries ${\mu }_{FM\mp }$ correspond to topological transitions signalled by the closing of the QP gap as seen in Fig. 1b. Thus, the topological transitions at *μ*_*FM*±_ coincide essentially with Lifshitz transitions in the electronic bands of the FM wire. Note that the parameters in our numerical treatment imply no overlap of the up and down spin bands. The QP wave function of the particle-hole symmetric eigenstates at zero energy is displayed in Fig. 1c for *μ*_*FM*_ = *h*_*z*_. We observe that these zero energy states are localized at the two edges of the FM wire (the white rectangle defines the wire), whereby the left (right) panel depicts the spin down (up) component. These bound states clearly correspond to a pair of MFs localized on each edge of the wire as confirmed by Fig. 1d which depicts the same zero energy eigenstate projected in the Majorana basis (see Supplementary Material). Since this is the only zero energy eigenstate separated by a finite energy gap from the continuum and the FM polarization is assumed to be *up* by convention here, the *right* panel of Fig. 1c may correspond to the zero-bias normalized conductance maps revealed by STM measurements in which FM and STM tip polarizations are the same^25^ or to those in which the STM tip is not polarized^29^ and both spin orientations contribute. On the other hand, the peculiar *“double-eye”* structure at the edges in the *left* panel of Fig. 1c may also be observable in some zero bias normalized conductance maps of spin polarized STM with sufficiently high resolution^30^ identifying the wires supporting MFs that have FM polarization opposite to that of the STM tip.

The origin of this behavior lies in the interplay between the different fields cooperating in the Hamiltonian and can be understood with the scheme of the *quartet rules* put forward by one of the authors^28^. According to these rules *four* fields (operators) form a *quartet*, if their matrix representations $\hat{A},\hat{B},\hat{C}$ and $\hat{D}$ obey the relation: $\hat{A}\hat{B}\hat{C}\hat{D}=\,\pm \,\,\hat{1}$ ^28^. As a consequence, the presence of any set of three members of a quartet implies that the missing fourth member is intrinsically generated, a phenomenon named the *quartet rule coupling* between the fields^28^. For example, the combination of charge and spin density wave (CDW and SDW) together with a chemical potential ensuring electron-hole asymmetry can give rise to a ferromagnetic spin polarization, important in the context of colossal magnetoresistance^31^. Another quartet case has been considered for unconventional superconductors with *d*-wave pairing combined with a SDW state which in conjunction with electron-hole asymmetry yields a so-called staggered *π*-triplet superconducting phase^32^, as might be realized in the puzzling high-field low-temperature Q-phase of CeCoIn_5_^33^.

**Figure 2:**
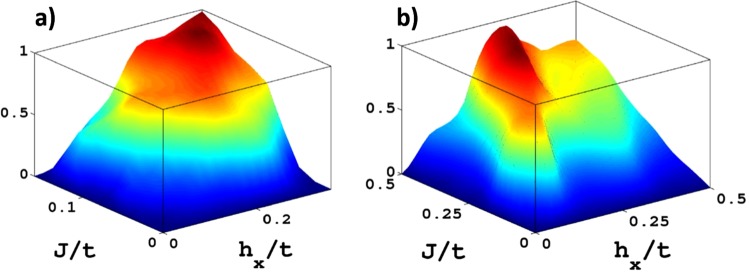
Quartet rule coupling^28^ for quartets A and B. (**a**) Induced spin-orbit-coupling (SOC) normalized to its maximal value as a function of the charge current and the Zeeman field in the presence of finite chemical potential producing particle-hole asymmetry. (**b**) The same for induced *p*-wave superconductor (SC) in the presence of conventional s-wave superconductor. Note that only when *both* the current and the Zeeman field are non zero, the quartet rule coupling applies and we have the induced SOC and *p*-wave SC fields confirming quartets A and B respectively (see Table 1 and Supplement I).

Two such quartets are relevant in our model, and are specially suitable for engineering of MFs: quartet A composed of charge current, Zeeman field, electron-hole asymmetry and antisymmetric SOC and quartet B with charge current, Zeeman field, conventional singlet SC and *p*-wave triplet SC. Both quartets share the first two fields, but differ in the other two. We use the basic symmetries inversion ${\mathcal I}$, time reversal ${\mathscr{T}}$ and their combination ${\mathcal R} = {\mathcal I} {\mathscr{T}}$ to characterize the fields of the quartets as being even (+) or odd (−) (see table). In terms of these symmetries electron-hole asymmetry and conventional SC behave equivalently as well as the pair SOC and triplet SC. In case A the quartet rule implies that in a system with electron-hole asymmetry the presence of a charge current ***J*** and a Zeeman field ***h*** induces SOC of the kind $(\hat{{\boldsymbol{J}}}\cdot {{\boldsymbol{g}}}_{{\boldsymbol{i}},{\boldsymbol{j}}})(\hat{{\boldsymbol{h}}}\cdot \tilde{{\boldsymbol{\sigma }}})$ with $\hat{{\boldsymbol{J}}},\hat{{\boldsymbol{h}}}$ unitary vectors along ***J***, ***h***, as is verified within our model and displayed in Fig. 2a. In the very same way we see that charge current, Zeeman field and conventional SC drives a spin triplet p-wave component with the real-space structure $\hat{{\boldsymbol{J}}}\cdot {{\boldsymbol{g}}}_{{\boldsymbol{i}},{\boldsymbol{j}}}{\tau }_{1}(i{\sigma }_{2})(\hat{{\boldsymbol{h}}}\cdot \tilde{{\boldsymbol{\sigma }}})$ (Fig. 2b).

**Table 1:** The quartets A and B and the parity of each of the involved fields under the basic symmetry operations of inversion ${\mathcal I}$, time reversal ${\mathscr{T}}$ and their combination ${\mathcal R} = {\mathcal I} {\mathscr{T}}$.

Quartet A	${\boldsymbol{ {\mathcal I} }}$	${\mathscr{T}}$	${\boldsymbol{ {\mathcal R} }}$	Quartet B	${\boldsymbol{ {\mathcal I} }}$	${\mathscr{T}}$	${\boldsymbol{ {\mathcal R} }}$
charge current	−	−	+	charge current	−	−	+
Zeeman field	+	−	−	Zeeman field	+	−	−
electron-hole asymmetry	+	+	+	conventional SC	+	+	+
spin-orbit coupling	−	+	−	triplet *p*-wave SC	−	+	−

Whenever three members of a quartet are present the fourth member is induced^28^.

A detailed analysis of the numerical results on Hamiltonian (1) provides insight into the key role of quartet rule coupling between fields. Besides the creation of the spin triplet component ${{\rm{\Delta }}}_{y}^{p}{{\boldsymbol{g}}}_{{\boldsymbol{i}},{\boldsymbol{j}}}\cdot \hat{{\boldsymbol{x}}}{\tau }_{2}$ through the presence of charge current, Zeeman field ${h}_{y}={\boldsymbol{h}}\cdot \hat{{\boldsymbol{y}}}$ and the spin-singlet pairing component, the Zeeman field component ${h}_{z}={\boldsymbol{h}}\cdot \hat{{\boldsymbol{z}}}$ combines with the spin-triplet pairing field ${{\rm{\Delta }}}_{y}^{p}{{\boldsymbol{g}}}_{{\boldsymbol{i}},{\boldsymbol{j}}}\cdot \hat{{\boldsymbol{x}}}{\tau }_{2}$ and particle-hole asymmetry to induce $\Im {{\rm{\Delta }}}_{x}^{p}{{\boldsymbol{g}}}_{{\boldsymbol{i}},{\boldsymbol{j}}}\cdot \hat{{\boldsymbol{x}}}{\tau }_{2}{\sigma }_{3}$ where ${{\rm{\Delta }}}_{y}^{p}$ ($\Im {{\rm{\Delta }}}_{x}^{p}$) are even(odd) under time-reversal. This results from the quartet D discussed in Supplement I.

This combination of triplet pairing fields eventually constitutes the basis of the Kitaev spinless model^9^. Based on this it is also possible now to establish qualitatively the *character* of the topological phase transition (TPT) suggested by Fig. 1b, using an effective 1D Hamiltonian for the FM wire that contains all the fields induced by the quartet rule,

2 $$\begin{array}{rcl}{H}_{FM}^{eff} & = & \sum _{i}\,{{\rm{\Psi }}}_{i}^{\dagger }[(t^{\prime} {f}_{i,j}^{x}+{\mu }_{FM}{\delta }_{i,j}){\tau }_{3}-{h}_{z}{\delta }_{i,j}{\tau }_{3}{\sigma }_{3}\\  &  & +\,{\rm{\Delta }}^{\prime} {\delta }_{i,j}{\tau }_{2}{\sigma }_{2}+{g}_{i,j}^{x}({\alpha }_{y}{\tau }_{3}{\sigma }_{2}+{{\rm{\Delta }}}_{y}^{p}{\tau }_{2}+\Im {{\rm{\Delta }}}_{x}^{p}{\tau }_{2}{\sigma }_{3})]{{\rm{\Psi }}}_{j}.\end{array}$$

with *t*′ the renormalized hopping matrix element^34^ with ${f}_{i,j}^{x}={\delta }_{j,i\pm 1}$, Δ′ the singlet pairing component induced by proximity and *α*_*y*_ the effective SOC appearing through the quartet rule combining charge current, Zeeman field and electron-hole asymmetry^28^.

Hamiltonian Eq. 2 belongs to the chiral BDI symmetry class (details are in the Supplement III) which for 1D accepts a strong integer ${\mathbb{Z}}$ topological invariant^35^. The system is in a non-trivial topological phase with a single pair of zero energy Majorana modes, when $\mathrm{|2}t^{\prime} -\sqrt{{({h}_{z})}^{2}-{\Delta }^{^{\prime} 2}}| < |{\mu }_{FM}| < \mathrm{|2}t^{\prime} +\sqrt{{({h}_{z})}^{2}-{\Delta }^{^{\prime} 2}}|$ (see Supplement III) that identifies the chemical potential range for which a single energy band is partially occupied. We conclude that the non-trivial topological region in Fig. 1b indicates *t*′ ≈ 0.6*t* and is almost symmetric with respect to *μ*_*FM*_ = *h*_*z*_ = 4 because Δ′ is rather small.

**Figure 3:**
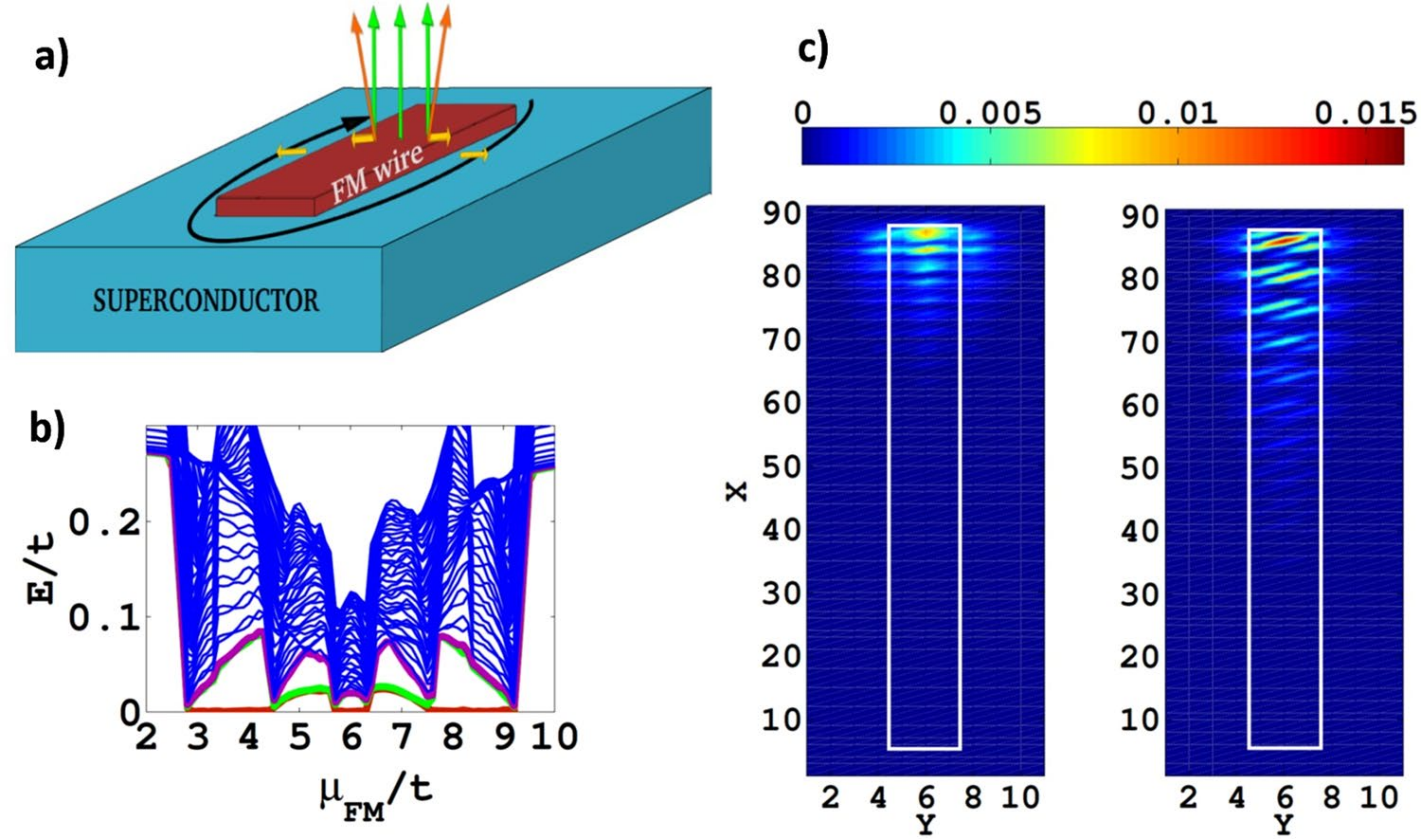
Quasi-one dimensional wire. (**a**) The finite width *W* quasi-1D FM wire with eventual tilting of the magnetization. Here as well flipping the sign of the perpendicular field and/or of the supercurrent and/or that of all in plane fields leaves the results invariant. (**b)** Typical low-energy quasiparticle spectrum for Δ = 1, *μ*_*SC*_ = 0, *h*_*z*_ = 6, |*h*_*y*_| = 0.8, |*J*| = 0.2, (all in t units) and magnetization ${\hat{h}}_{1}={\hat{h}}_{z}+{\hat{h}}_{y}$, ${\hat{h}}_{2}={\hat{h}}_{z}$ and ${\hat{h}}_{3}={\hat{h}}_{z}-{\hat{h}}_{y}$ for the first, second and third row respectively of this *W* = 3 wire. We observe that a single near zero eigenenergy (red line) emerges when odd numbers of transverse sub-bands in the wire are partially occupied e.g. near *μ*_*FM*_/*t* = 4 (1 sub-band) and near *μ*_*FM*_/*t* = 6 (three sub-bands) as anticipated^36^. Near *μ*_*FM*_/*t* = 5 two transverse sub-bands cross the Fermi level and the two pairs of MFs interfere acquiring finite energy (green line). (**c)** One Majorana mode for *μ*_*FM*_/*t* = 4 (left panel) and one for *μ*_*FM*_/*t* = 6 (right panel). The *μ*_*FM*_/*t* = 6 Majorana mode is less localized because it is protected by a smaller energy gap from the lowest eigenenergy of the continuum (purple lines) than the *μ*_*FM*_/*t* = 4 mode. The white rectangle defines the FM wire.

To illustrate the robustness of these Majorana modes, we extend our discussion to a FM wire of finite width *W*, still small compared to the length *L*, incorporating a possible tilting of the magnetic moment in the wire as indicated in Fig. 3a. The results of our numerical analysis are shown in Fig. 3b,c where the finite *W* corresponds to 3 lattice sites introducing three bands in the FM wire which are spin split. In Fig. 3c are shown only one of the two MF modes obtained for each of the two topological regimes reached for *μ*_*FM*_ = 4 (left panel) and *μ*_*FM*_ = 6 (right panel) respectively.

The multiple TPTs in Fig. 3b yield topologically non-trivial phase in the range of *μ*_*FM*_, where the FM wire has an odd number of partially filled bands that could host Cooper pairing, which again is connected with Lifshitz transitions. Additionally we notice that the finite width *W* allows now for transverse spin triplet pairing, i.e. a field of the type ${g}_{{\boldsymbol{i}},{\boldsymbol{j}}}^{y}{\tau }_{1}$ which combines with the component ${g}_{{\boldsymbol{i}},{\boldsymbol{j}}}^{x}{\tau }_{2}$ to a Cooper pair with chiral symmetry (“*p*_*x*_ ± *ip*_*y*_”) (quartet D in Supplement I). This phase belongs, thus, to the symmetry class D with a ${{\mathbb{Z}}}_{2}$ topological invariant^35^. As elaborated in refs^36,37^, for $W\lesssim \xi$, where $\xi \propto \frac{t}{{{\rm{\Delta }}}_{{p}_{y}}}$ is the coherence length of the transverse SC component *p*_*y*_, the D symmetry class yields a pair of zero-energy MFs, if an *odd* number of transverse sub-bands are partially occupied. This is the case in our model calculations with W = 3 (see Fig. 3), where indeed the coherence length of the emergent transverse *p*_*y*_ SC is greater than the transverse dimension W.

**Figure 4:**
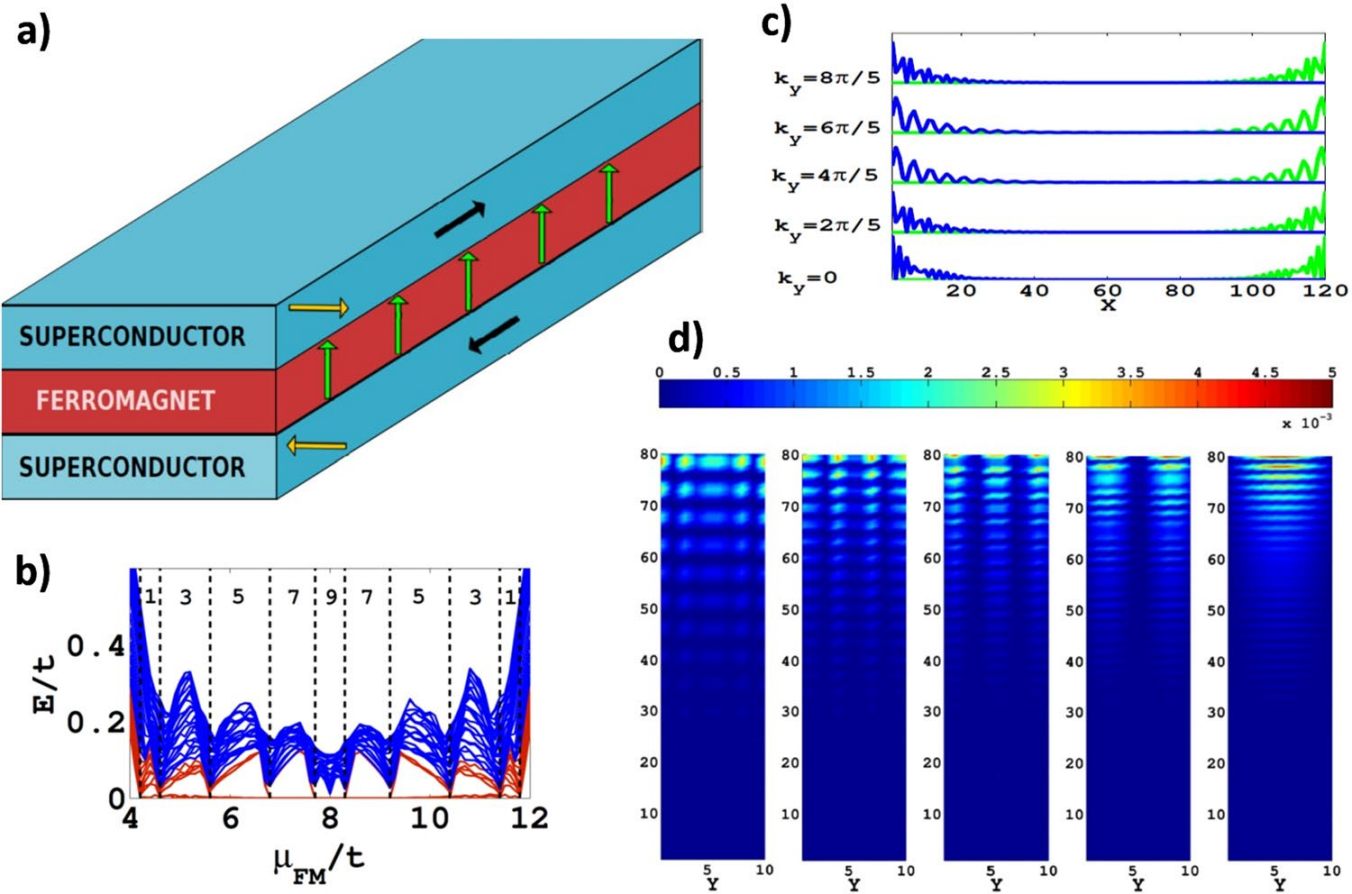
Layered heterostructure for controllable generation of Majorana fermions. (**a**) SC/FM/SC trilayer with antiparallel supercurrents (black arrows) and Zeeman fields (yellow arrows), perpendicular to the FM magnetization. Provided supercurrents and in-plane fields in the adjacent SC layers remain antiparallel, the signs of fields and currents has no influence on the results. Moreover, provided green and yellow Zeeman fields are in perpendicular directions, the exact direction of these fields is irrelevant. (**b**) Typical low-energy quasiparticle spectrum for Δ = 4, *μ*_*SC*_ = 0, *h*_*z*_ = 8, |*h*_*y*_| = 2, |*J*| = 0.6 and *t*_*c*_ = 0.8 for the interlayer hopping term all in units normalized to the in-plane hopping term *t*. Here we have *N*_*x*_ = 120, *N*_*y*_ = 10 and *periodic* boundary conditions along y-axis. With red lines we denote the branches which pin to zero energy for some *μ*_*FM*_ values. Dashed lines indicate the topological phase transitions while the numbers on top correspond to the value of the topological invariant ${\mathscr{W}}$ (see Supplements II and III). (**c**) The five pairs of Majorana fermions for *μ*_*FM*_/*t* = 6 corresponding to the ${\mathscr{W}}=5$ regime in (**b**). (**d)** One Majorana fermion from each of the five Majorana fermion pairs that we obtain for the same parameters but with *open* boundary conditions along y-axis. The system remains manifestly in a BDI symmetry class for both types of boundary conditions.

After the discussion of MFs in the embedded FM wire we turn to a further related design which might be more suitable for practical MF engineering. It is important to note that the structure of the device needs not to be one-dimensional, as in the above device, but that applied currents are sufficient to establish the necessary directionality. As an example of this kind of device we present here a three-layer structure (see Fig. 4a) which consists of a FM layer sandwiched between two conventional SCs. The FM magnetization (green arrows) here points perpendicular to the layer and the adjacent SC layers carry supercurrents in opposite directions (black arrows) and in-plane Zeeman fields in opposite directions as well (yellow arrows). The corresponding model Hamiltonian for our numerical analysis is given by

3 $$\begin{array}{rcl} {\mathcal H}  & = & \sum _{{\boldsymbol{i}},{\boldsymbol{j}},l,l^{\prime} }\,{{\rm{\Psi }}}_{{\boldsymbol{i}},l}^{\dagger }\,[[({\mu }_{l}{\tau }_{3}-{\tau }_{3}{{\boldsymbol{h}}}_{l}\cdot \tilde{{\boldsymbol{\sigma }}}+{{\rm{\Delta }}}_{l}{\tau }_{2}{\sigma }_{2}){\delta }_{{\boldsymbol{i}},{\boldsymbol{j}}}\\  &  & +\,{t}_{l}{f}_{{\boldsymbol{i}},{\boldsymbol{j}}}{\tau }_{3}+{{\boldsymbol{J}}}_{l}\cdot {{\boldsymbol{g}}}_{{\boldsymbol{i}},{\boldsymbol{j}}}]{\delta }_{l,l^{\prime} }+{t}_{l,l^{\prime} }{\tau }_{3}]{{\rm{\Psi }}}_{{\boldsymbol{j}},l^{\prime} },\end{array}$$

where *l* is a layer index and *t*_*l*,*l*′_ the interlayer hopping term. The numerical results for such a system of three layers are shown in Fig. 4b–d.

Again we see a sequence of TPTs between states involving different number of MF pairs upon changing the chemical potential *μ*_*FM*_ in the FM layer (Fig. 4b). Although in the particular case demonstrated in Fig. 4b only odd number of MFs pairs emerge, in general, also topological phases with even topological invariant can also be reached (Supplement II).

The TPTs of Fig. 4b are understood qualitatively from an effective Hamiltonian for the FM layer corresponding to our numerical findings that should exhibit a *parallel rows* structure:

4 $${ {\mathcal H} }_{FM}^{eff}=\sum _{i,j,\nu ,\nu ^{\prime} }\,{{\rm{\Psi }}}_{i,\nu }^{\dagger }[{H}_{i,j,\nu }^{1D}{\delta }_{\nu ,\nu ^{\prime} }+{t^{\prime} }_{\perp }{\tau }_{3}{\sigma }_{0}{\delta }_{\nu ^{\prime} ,\nu \pm 1}{\delta }_{i,j}]{{\rm{\Psi }}}_{j,\nu ^{\prime} }$$

Each row along the x-axis of the FM layer is indexed with *ν* and *t*′_⊥_ is the renormalized transverse inter-row hopping term along the y-axis. The 1D Hamiltonian ${H}_{i,j,\nu }^{1D}$ has exactly the same form as Eq. 2.

The system is translationally symmetric along the transverse direction when periodic boundary conditions apply while for open boundary conditions it only maintains the reflection symmetry. In either case, the Hamiltonian Eq. 4 takes a block diagonal form (Supplement II)

5 $${ {\mathcal H} }_{FM}^{eff}=\sum _{i,j,n}\,{{\rm{\Psi }}}_{i,n}^{\dagger }[{H}_{i,j,n}^{1D}+{\tau }_{3}{\sigma }_{0}{\lambda }_{n}{\delta }_{i,j}]{{\rm{\Psi }}}_{j,n},$$

where *λ*_*n*_ are the eigenvalues of matrix *H*_⊥_ = *t*′_⊥_*δ*_*ν*′,*ν*±1_. Therefore, the system belongs to the $BDI{\oplus }^{{N}_{y}}$ class with the integer topological invariant ${\mathscr{W}}={\sum }_{n}\,{{\mathscr{W}}}_{n}$. Since *λ*_*n*_ act as an effective chemical potential which breaks the degeneracy of the 1D sub-systems^38^, the topological criteria for *W*_*n*_ = 1 are modified accordingly: $\mathrm{|2}t^{\prime} -\sqrt{{h}_{z}^{2}-{{\rm{\Delta }}}^{^{\prime} 2}}| < |\mu +{t^{\prime} }_{\perp }{\lambda }_{n}\mathrm{| < |2}t^{\prime} +\sqrt{{h}_{z}^{2}-{{\rm{\Delta }}}^{^{\prime} 2}}|$. For periodic boundary conditions when *t*′ ≈ *t*′_⊥_ and *N*_*y*_ is even, only odd values of *W* are observed as presented in Fig. 4b corresponding to Majorana multiplets obeying non-Abelian statistics. For open boundary conditions the residual degeneracy of the transverse bands is lifted and transitions among topological phases with odd and even number of MFs pairs are observed (Supplement II). We note that the results and discussions presented here are based on a single FM layer, however this is not a necessary condition as will be discussed in a future work.

To conclude, we have identified *quartets* of fields that are opening novel extraordinary paths for the quantum engineering of MFs in conventional SC/FM heterostructures. No exotic materials with special structures of intrinsic Rashba SOC are needed. These *quartets* of fields have been deliberately discussed here only in the context of MF engineering in FM/SC heterostructures. Their broader implications in a variety of other phenomena will be explored elsewhere.

## Supplementary information

Supplementary Online Material for: \\ Alternative paths to realize Majorana Fermions in Superconductor-Ferromagnet Heterostructures
