# Supplementary material for: Alternative paths to realize Majorana Fermions in Superconductor-Ferromagnet Heterostructures
Source: Sci Rep. 2019 Apr 18;9:6259. doi: 10.1038/s41598-019-42558-3 (PMC6472391; doi:10.1038/s41598-019-42558-3)
Supplement: Supplementary file 1 — Supplementary Online Material for: \\ Alternative paths to realize Majorana Fermions in Superconductor-Ferromagnet Heterostructures [file 41598_2019_42558_MOESM1_ESM.pdf]

Supplementary Online Material for: Alternative paths to realize Majorana Fermions in Superconductor-Ferromagnet Heterostructures

G. Livanas[1], G. Varelogiannis[1] and M. Sigrist[2]

[1]Department of Physics, National Technical University of Athens, GR-15780 Athens, Greece
[2] Institut für Theoretische Physik, ETH Zürich, 8093 Zürich, Switzerland

I. QUARTETS INVOLVED AND INDUCED FIELDS

In previous work by one of us, it was demonstrated that four fields or order parameters with respective matrix representations for example $\widehat{A}$, $\widehat{B}$, $\widehat{C}$ and $\widehat{D}$ that obey the *quartet rule*: $\widehat{A}\widehat{B}\widehat{C}\widehat{D} = \pm\widehat{1}$ form a *quartet* meaning that *the presence of any three of them generates the missing fourth one* via what was named the *quartet rule coupling* [1]. Note that the rule holds as well for all cyclic permutations of the matrix representations in the product. The phenomenon of *quartet rule coupling* has also been proven numerically on dozens of different examples over the last years and there are many cases where *quartets* extracted from the above *rule* have been shown to provide significant insight into various puzzling problems of correlated electron systems especially when multiple order parameters or fields are involved [2]. Here we focus on the *quartets* that are relevant for our Majorana engineering approach illustrating how the phenomenon of *quartet rule coupling* manifests there.

More precisely, in this section we demonstrate how triplet superconducting (SC) p fields and spin-orbit coupling (SOC) are induced when a Zeeman field $\boldsymbol{h}$ and a charge current J couple to a conventional singlet SC field Δ and a chemical potential μ respectively. For simplicity we consider a 1D system though it is straightforward to extent the same results to systems with arbitrary dimensions and lattice symmetry. The Hamiltonian for the particular fields acquires the following form in coordinate space.

$$\mathcal{H} = \frac{1}{2}\sum_{i,j} \Psi_i^\dagger \left[(\mu\tau_3 + \Delta\tau_2\sigma_2 - h\tau_3\boldsymbol{d}_h\cdot\tilde{\boldsymbol{\sigma}})\delta_{i,j} + t\delta_{j,i\pm1}\tau_3 \pm iJ\delta_{j,i\pm1} \right] \Psi_j \, , \tag{S1}$$

where we have introduced the spin dependent Nambu spinor $\Psi_i^\dagger = \left(\psi_{i,\uparrow}^\dagger, \psi_{i,\downarrow}^\dagger, \psi_{i,\uparrow}, \psi_{i,\downarrow}\right)$ and the Pauli matrices $\boldsymbol{\tau}$ and $\boldsymbol{\sigma}$ acting on Nambu and spin space respectively. With $\boldsymbol{d}_h$ we denote the vector of the Zeeman field in spin space, i the coordinate along the x- coordinate axis, while $\tilde{\boldsymbol{\sigma}} = (\sigma_1, \tau_3\sigma_2, \sigma_3)$. In Table I we demonstrate how the terms of the Hamiltonian transform under the inversion symmetry operation $\mathcal{I}$ acting in coordinate space, the time reversal symmetry operation $\mathcal{T}$ effected by the anti-unitary operator $\Theta = i\sigma_2\mathcal{K}$, where $\mathcal{K}$ acts as complex-conjugation and the combined symmetry operation $\mathcal{R} = \mathcal{I}\mathcal{T}$.

According to [1], the charge current J and the Zeeman field $h\boldsymbol{d}_h$ couple to a chemical potential μ and a conventional SC field Δ to induce SOC of the form $\pm i\alpha\boldsymbol{d}_h\cdot\tilde{\boldsymbol{\sigma}}\delta_{j,i\pm1}$ and the triplet p SC field $\pm i\Delta^p\tau_1\boldsymbol{d}_h\cdot i\sigma_2\tilde{\boldsymbol{\sigma}}\delta_{j,i\pm1}$ respectively. Indeed, matrix representations of those fields $\widehat{A}, \widehat{B}, \widehat{C}$ and $\widehat{D}$ obey the *quartet coupling rule* $\widehat{A}\widehat{B}\widehat{C}\widehat{D} = \pm\widehat{1}$ [1] forming the overlapping quartets A and B (see table I) that have common elements the charge current and the Zeeman field. The spin space vector of the induced triplet fields is determined by the Zeeman field while their spatial configuration by the charge current.

Regarding the symmetry operations considered above SOC is even under $\mathcal{T}$ and odd under $\mathcal{I}$ and $\mathcal{R}$. Considering a charge current J, a Zeeman field $h\boldsymbol{d}_h$ and a chemical potential μ we observe that their product corresponds to a field with the same behaviour under these three symmetry operations as SOC. Therefore when the above three fields coexist SOC is effectively induced. A triplet SC field has the same transformation properties as SOC. However in addition pairing fields lock the charge conjugation symmetry operation. Therefore triplet SC fields are effectively induced when a charge current J and a Zeeman field $h\boldsymbol{d}_h$ coexist with a conventional SC field instead of a chemical potential. Notice that according to [1] every field is equivalent to a product of *at least* three distinct fields.

While the validity of the quartet coupling rule [1] is generic, to illustrate how this coupling emerges in quartets A and B we present analytical results considering a 1D translationally invariant system for the particular case where $\boldsymbol{d}_h = (1, 0, 0)$ for the Zeeman field. In momentum space the Hamiltonian acquires the following form

Quartet A		$\mathcal{I}$	$\mathcal{T}$	$\mathcal{R}$
Charge current	$\pm iJ\delta_{j,i\pm1}$	$-$	$-$	$+$
Zeeman field	$h\tau_3\boldsymbol{d}_h\cdot\tilde{\boldsymbol{\sigma}}\delta_{j,i}$	$+$	$-$	$-$
Chemical potential	$\mu\tau_3\delta_{j,i}$	$+$	$+$	$+$
spin-orbit coupling	$\pm i\alpha\boldsymbol{d}_h\cdot\tilde{\boldsymbol{\sigma}}\delta_{j,i\pm1}$	$-$	$+$	$-$

Quartet B		$\mathcal{I}$	$\mathcal{T}$	$\mathcal{R}$
Charge current	$\pm iJ\delta_{j,i\pm1}$	$-$	$-$	$+$
Zeeman field	$h\tau_3\boldsymbol{d}_h\cdot\tilde{\boldsymbol{\sigma}}\delta_{j,i}$	$+$	$-$	$-$
Conventional SC	$\Delta\tau_2\sigma_2\delta_{j,i}$	$+$	$+$	$+$
Triplet p SC	$\pm i\Delta^p\tau_1\boldsymbol{d}_h\cdot i\sigma_2\tilde{\boldsymbol{\sigma}}\delta_{j,i\pm1}$	$-$	$+$	$-$

TABLE I: (left) Quartet A : Induced spin-orbit coupling by combining a charge current J with a chemical potential μ and a Zeeman field $h\boldsymbol{d}_h$ where $\boldsymbol{d}_h$ is a vector in spin space. The particular fields transform symmetrically(anitsymmetrically), denoted by $+(-)$, under the symmetry operations of inversion $\mathcal{I}$ $(i\leftrightarrow j)$, time reversal $\mathcal{T}$ $(\Theta = i\sigma_2\mathcal{K})$ and the combined operation $\mathcal{R}=\mathcal{IT}$. In order for spin-orbit coupling to be induced in the presence of a charge current $\mathcal{T}$ and $\mathcal{R}$ have to break by the application of a chemical potential and a Zeeman field respectively. (right) Quartet B : In the case of induced triplet p SC field the symmetry arguments are the same if instead of the chemical potential we consider a conventional SC field Δ.

$$\mathcal{H}=\frac{1}{2}\sum_k \Psi_k^\dagger\left[(\mu+\epsilon(k))\tau_3+\Delta\tau_2\sigma_2+J(k)-h_x\tau_3\sigma_1\right]\Psi_k\,, \tag{S2}$$

where we have introduced the spinor $\Psi_k^\dagger=\left(c_{k,\uparrow}^\dagger,c_{k,\downarrow}^\dagger,c_{-k,\uparrow},c_{-k,\downarrow}\right)$ and the Pauli matrices $\boldsymbol{\tau}$ and $\boldsymbol{\sigma}$ acting on Nambu and spin space respectively. We have also added a kinetic term $\epsilon(k)=\epsilon(-k)$ present in most realistic models, while for the current term by definition we have $J(k)=-J(-k)$. The equal spin pairing P_x and the spin-orbit coupling A_x correlations are calculated through the following equations.

$$P_x=\frac{1}{2N}\sum_{k,i\omega_n} f_{P_x}(k)Tr\{\tau_1\sigma_3\boldsymbol{G}(k;i\omega_n)\}=\frac{1}{2N}\sum_{k,n} f_{P_x}(k)[U(k)^\dagger\tau_1\sigma_3U(k)\ ]_{nn}n_F(E_n(k))\rightarrow$$
$$P_x=\frac{1}{N}\sum_{k,m,s} f_{P_x}(k)\frac{[m\cdot s]\Delta}{\sqrt{\Delta^2+[\mu+\epsilon(k)]^2}}n_F(E_{m,s}(k))\,, \tag{S3}$$

$$A_x=\frac{1}{2N}\sum_{k,i\omega_n} f_{A_x}(k)Tr\{\sigma_1\boldsymbol{G}(k;i\omega_n)\}=\frac{1}{2N}\sum_{k,n} f_{A_x}(k)[U(k)^\dagger\sigma_1U(k)\ ]_{nn}n_F(E_n(k))\rightarrow$$
$$A_x=\sum_{k,m,s}\frac{-[m\cdot s]f_{A_x}(k)(\mu+\epsilon(k))}{N\sqrt{\Delta^2+[\mu+\epsilon(k)]^2}}n_F(E_{m,s}(k)) \tag{S4}$$

where N the number of the lattice points of the system or equivalently the number of momenta k within the first Brillouin zone, n=1-4 corresponding to the dimension of the spinor, $m,s=\pm$, $\boldsymbol{G}(k;i\omega_n)$ the momentum space Matsubara Green functions defined as $\boldsymbol{G}(k;i\omega_n)=[i\omega_n-H_k]^{-1}$, and $\hat{U}(k)^\dagger=exp\left[i\pi/4\tau_1\sigma_2\right]exp\left[i\pi/4\tau_2\right]$ the unitary transformation which diagonalises the Hamiltonian matrix for each momentum $\hat{U}(k)^\dagger\mathcal{H}\hat{U}(k)=E_{m,s}(k)$, with $E_{m,s}(k)=J(k)-sh_x+m\sqrt{\Delta^2+[\mu+\epsilon(k)]^2}$, the excitation spectrum. The momentum configuration functions for both terms are antisymmetric $f_{A_x}(k)=f_{A_x}(-k)$, $f_{P_x}(k)=f_{P_x}(-k)$ as imposed by the anticommutation rules for fermions. Notice that the induced equal spin pairing correlations and spin-orbit coupling are of the particular representation $\tau_1\sigma_3$ and σ_1 respectively and in fact are the *only* induced correlations, as *only* for these representations $\hat{O}$ the matrix $[\hat{U}(k)\hat{O}\hat{U}(k)^\dagger]$ acquires non-zero diagonal elements, or equivalently the $Tr\{\hat{O}\boldsymbol{G}(k;i\omega_n)\}$ can be finite. Indeed we have $[\hat{U}(k)\tau_1\sigma_3\hat{U}(k)^\dagger]=\tau_3\sigma_3$, while $[\hat{U}(k)\sigma_1\hat{U}(k)^\dagger]=-\tau_3\sigma_3$, leading to the final form of the induced correlations for the particular system. From Eq. S3 it is straightforward that $P_x=0$ when $\Delta=0$. However only when also $h_x\neq0$ and $J\neq0$, finite P_x correlations are induced, since for $h_x=0$ we get $n_F(E_{m,+}(k))=n_F(E_{m,-}(k))\rightarrow P_x=0$ and for J=0 again we have $n_F(E_{m,s}(k))=n_F(E_{m,s}(-k))\rightarrow P_x=0$ due to $f_{P_x}(k)=-f_{P_x}(-k)$. Based on similar considerations we can show that spin-orbit coupling correlations $A(x)$ become finite only when $J\neq0$, $\mu\neq0$ and $h_x\neq0$ while the momentum configuration functions $f_{A_x}(k)$ is determined again by the corresponding function $f_J(k)$ for the current. In Fig. S1 we demonstrate how P_x triplet equal spin pairing correlations and A_x spin-orbit coupling are induced for particular values of the singlet pairing field Δ and the chemical potential μ as the charge current J and the Zeeman field h_x increase. Finally we have to remark that the induced correlations and their conjugate fields vanish when the momentum configuration functions $f_{P_x}(k)(f_{A_x}(k))$ for $P_x(A_x)$ and $f_J(k)$ for the charge current J, belong to different irreducible representations of the corresponding point symmetry group of the system.

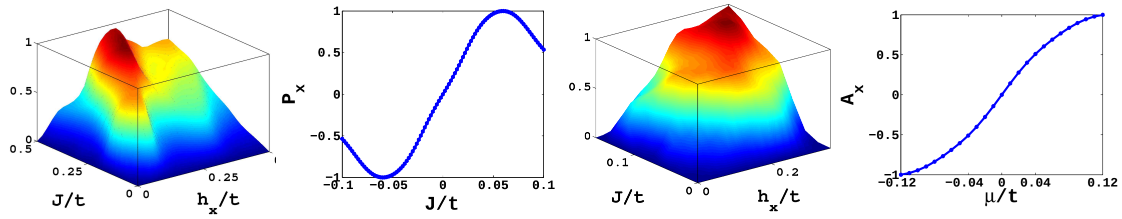

(a) (b) (c) (d)

FIG. S1: a) For singlet pairing field $\Delta = 0.5t$ and chemical potential $\mu = 0$ the induced $P_x\tau_1\sigma_3$ correlations as the charge current J and the Zeeman field h_x increase. The induced P_x correlations are normalized with respect to the highest value for this range of parameters. b) For the same parameters and $h_x = 0.1t$ the induced P_x correlations with respect to charge current J. Notice that the sign of the induced correlations is determined by the direction of the current. c) For chemical potential $\mu = 0.5t$ the induced $A_x\sigma_1$ spin-orbit coupling correlations for increasing values of the charge current J and the Zeeman field h_x. d) The induced A_x correlations with respect to the chemical potential μ for current $J = 0.2t$ and $h_x = 0.4t$. The sign of the induced A_x correlations is determined by the chemical potential.

Using the same symmetry arguments we anticipate triplet odd in time reversal $\pm i\Im\Delta^p\tau_2\boldsymbol{d}_{\Im p}\cdot i\sigma_2\tilde{\boldsymbol{\sigma}}\delta_{j,i\pm\boldsymbol{x}}$ SC fields to emerge in a conventional SC $\Delta\tau_2\sigma_2$ with SOC $\pm i\alpha\boldsymbol{d}_\alpha\cdot\tilde{\boldsymbol{\sigma}}\delta_{j,i\pm\boldsymbol{x}}$ when a Zeeman field $h\tau_3\boldsymbol{d}_h\cdot\tilde{\boldsymbol{\sigma}}$ is applied. As presented in Table. II SOC α and Zeeman fields h break the inversion $\mathcal{I}$ and time reversal $\mathcal{T}$ symmetries respectively in order for a triplet p SC field to be induced. In this case the vector $\boldsymbol{d}_{\Im p}$ in spin space of the induced p SC field derives from $\boldsymbol{d}_{\Im p} = \boldsymbol{d}_h\times\boldsymbol{d}_\alpha$ and therefore the three triplet fields must be orthogonal to each other, while the two SC fields acquire a $\pi/2$ relative phase. In a 1D translationally invariant system of a conventional SC with SO interactions $\alpha_x(k) = -\alpha_x(-k)$ under the effect of a Zeeman field $h_z\tau_3\sigma_3$, described by the following Hamiltonian

$$\mathcal{H} = \frac{1}{2}\sum_k \Psi_k^\dagger\left[\alpha_x(k)\sigma_1 + \Delta\tau_2\sigma_2 + h_z\tau_3\sigma_3\right]\Psi_k\,, \tag{S5}$$

the induced $\Im P_y\tau_2$ correlations derive from the following equation

$$\Im P_y = \sum_k\sum_{m,s} f_{\Im p_y}(k)[m\cdot s]\frac{\alpha_x(k)}{\sqrt{[\Delta + sh_z]^2 + [a_x(k)]^2}}n_F(E_{m,s}(k))\,, \tag{S6}$$

where $E_{m,s}(k) = m\sqrt{[\Delta + sh_z]^2 + [\alpha_x(k)]^2}$ the excitation spectrum of the system and $f_{\Im p_y}(k) = -f_{\Im p_y}(-k)$ the configuration function of the induced p correlations. From the above equation it is straightforward that $\Im P_y = 0$ when $a_x = 0$, or $\Delta = 0$ [as $n_F(E_{+,s}(k)) = n_F(E_{-,s}(k))$], or $h_z = 0$ [as $n_F(E_{m,+}(k)) = n_F(E_{m,-}(k))$].

In the same way triplet $\pm i\Im\Delta^p\tau_2\boldsymbol{d}_{\Im p}\cdot i\sigma_2\tilde{\boldsymbol{\sigma}}\delta_{j,i\pm\boldsymbol{x}}$ SC fields are anticipated to emerge when a Zeeman field $h\tau_3\boldsymbol{d}_h\cdot\tilde{\boldsymbol{\sigma}}$ and a chemical potential μ are applied to a triplet $\pm i\Delta^p\tau_1\boldsymbol{d}_p\cdot i\sigma_2\tilde{\boldsymbol{\sigma}}\delta_{j,i\pm\boldsymbol{x}}$ SC. The two SC fields acquire a relative phase $\phi = \pi/2$ and the vector $\boldsymbol{d}_{\Im p}$ in spin space of the induced field derives from $\boldsymbol{d}_{\Im p} = \boldsymbol{d}_p\times\boldsymbol{d}_h$. Particularly considering a 1D translationally invariant system of a equal spin triplet $\Delta_x^p(k)\tau_1\sigma_3$ SC under the effect of a Zeeman field $h_z\tau_3\sigma_3$ and a chemical potential μ, described by the following Hamiltonian

$$\mathcal{H} = \frac{1}{2}\sum_k \Psi_k^\dagger\left[\Delta_x^p(k)\tau_1\sigma_3 + \mu\tau_3 + h_z\tau_3\sigma_3\right]\Psi_k\,, \tag{S7}$$

the induced $\Im P_y\tau_1$ correlations derive from the following equation

$$\Im P_y = \sum_k\sum_{m,s} f_{\Im p_y}(k)[m\cdot s]\frac{\Delta_x^p(k)}{\sqrt{[\mu + sh_z]^2 + [\Delta_x^p(k)]^2}}n_F(E_{m,s}(k))\,, \tag{S8}$$

where $E_{m,s}(k) = m\sqrt{[\mu + sh^z]^2 + [p_x(k)]^2}$ the corresponding eigenvalues. Again $\Im P_y = 0$ when $\Delta_x^p = 0$, or $h_z = 0$ [as $n_F(E_{m,+}(k)) = n_F(E_{m,-}(k))$], or $\mu = 0$ [as $n_F(E_{+,s}(k)) = n_F(E_{-,s}(k))$].

Finally according to the quartet coupling rule of induced fields [1] the singlet pairing field $\Delta(\tau_2\sigma_2)$, the potential field $\mu(\tau_3)$, the Zeeman field $h\tau_3\boldsymbol{d}_h\cdot\tilde{\boldsymbol{\sigma}}$, spin-orbit interactions $\pm i\alpha\boldsymbol{d}_\alpha\cdot\tilde{\boldsymbol{\sigma}}\delta_{j,i\pm\boldsymbol{x}}$ and p SC fields $\pm i\Delta^p\tau_1\boldsymbol{d}_p\cdot i\sigma_2\tilde{\boldsymbol{\sigma}}\delta_{j,i\pm\boldsymbol{x}}$, $\pm i\Im\Delta^p\tau_2\boldsymbol{d}_{\Im p}\cdot i\sigma_2\tilde{\boldsymbol{\sigma}}\delta_{j,i\pm\boldsymbol{x}}$ form a closed pattern of coupled fields when $\boldsymbol{d}_{\Im p} = \boldsymbol{d}_\alpha\times\boldsymbol{d}_h = \boldsymbol{d}_p\times\boldsymbol{d}_h$ and $\boldsymbol{d}_\alpha = \boldsymbol{d}_p$. The pattern is closed in the sense that any subset of these fields will induce a field, if any, which belongs to the above set of six fields. A system with all these fields belongs to the BDI symmetry class as elaborated in Appendix III.

Quartet C		$\mathcal{I}$	$\mathcal{T}$	$\mathcal{R}$
Conventional SC	$\Delta\tau_2\sigma_2\delta_{j,i}$	$+$	$+$	$+$
Zeeman field	$h\tau_3\boldsymbol{d}_h\cdot\tilde{\boldsymbol{\sigma}}\delta_{j,i}$	$+$	$-$	$-$
SO interactions	$\pm i\alpha\boldsymbol{d}_\alpha\cdot\tilde{\boldsymbol{\sigma}}\delta_{j,i\pm1}$	$-$	$+$	$-$
Triplet $\Im p$ SC	$\pm i\Im\Delta^p\tau_2\boldsymbol{d}_{\Im p}\cdot i\sigma_2\tilde{\boldsymbol{\sigma}}\delta_{j,i\pm1}$	$-$	$-$	$+$

Quartet D		$\mathcal{I}$	$\mathcal{T}$	$\mathcal{R}$
Triplet p SC	$\pm i\Delta^p\tau_1\boldsymbol{d}_p\cdot i\sigma_2\tilde{\boldsymbol{\sigma}}\delta_{j,i\pm1}$	$-$	$+$	$-$
Zeeman field	$h\tau_3\boldsymbol{d}_h\cdot\tilde{\boldsymbol{\sigma}}\delta_{j,i}$	$+$	$-$	$-$
Chemical potential	$\mu\tau_3\delta_{j,i}$	$+$	$+$	$+$
Triplet $\Im p$ SC	$\pm i\Im\Delta^p\tau_2\boldsymbol{d}_{\Im p}\cdot i\sigma_2\tilde{\boldsymbol{\sigma}}\delta_{j,i\pm1}$	$-$	$-$	$+$

TABLE II: (left) Quartet C : Triplet p SC field is induced in a conventional SC Δ with SOC a when a Zeeman field h is applied. The singlet SC field Δ transforms symmetrically (denoted by $+$) under the symmetry operations of inversion $\mathcal{I}$ ($i \leftrightarrow j$), time reversal $\mathcal{T}$ ($\Theta = i\sigma_2\mathcal{K}$) and the combined operation $\mathcal{R} = \mathcal{I}\mathcal{T}$. An odd under $\mathcal{I}$ and $\mathcal{T}$ triplet p field is induced when SOC α and a Zeeman field h break $\mathcal{I}$ and $\mathcal{T}$ respectively. The induced p SC field acquires a $\pi/2$ phase with respect to the Δ conventional SC field, while its vector in spin space derives from $\boldsymbol{d}_{\Im p} = \boldsymbol{d}_h \times \boldsymbol{d}_\alpha$. (right) Quartet D : In the same way a triplet p SC field is induced in a $\pi/2$ relative phase with respect to another p SC field when a Zeeman field and a chemical potential are applied. The vector of the induced field is also determined by $\boldsymbol{d}_{\Im p} = \boldsymbol{d}_p \times \boldsymbol{d}_h$.

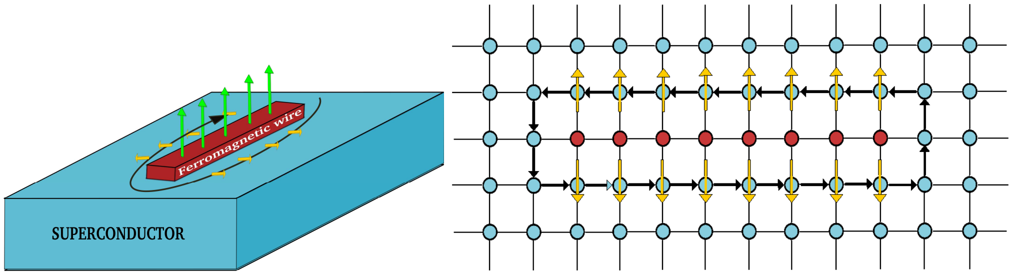

FIG. S2: *Left:* Ferromagnetic wire embedded in a conventional superconductor. The magnetization of the wire is considered perpendicular to the surface of the superconductor(green arrows). The magnetic field due to the magnetization of the wire is expected to stimulate a supercurrent flow (black arrow) and contribute an in-plane Zeeman field h_y (yellow arrows) with opposite polarization at the each side of the wire. *Right:* The effective 2-D lattice scheme on which our calculations are based (Hamiltonian (Eq. 1) in the Letter) describes a perpendicularly polarized 1D FM wire (red sites) embedded in a SC (blue sites). The magnetic field of the FM wire triggers a super-current flow in the SC (black arrows) and contributes to small in plane Zeeman field components (yellow arrows).

II. INDUCED FIELDS ON FERROMAGNET-SUPERCONDUCTOR HETEROSTRUCTURES

In this section we demonstrate how triplet SC correlations and SOC are induced in FM/SC heterostructures, due to a supercurrent flow J and a transverse to the magnetisation of the FM Zeeman field h.

a. 1D wire on a conventional SC First we consider the case of a 1D FM wire embedded in a conventional SC (Fig. S2). We assume the magnetisation of the FM wire perpendicular to the SC surface and the corresponding magnetic field B_z, triggers a supercurrent flow J in the SC region. Moreover the rotation of the magnetic field away from the FM wire contributes to a Zeeman field h_y component transverse to the magnetisation of the wire which is not screened by the supercurrent flow. Therefore in lattice points within the SC region and adjacent to the FM wire we consider a finite supercurrent flow and a Zeeman field h_y as depicted in Fig. S2. For the SC region we consider a finite on-site SC field Δ and we choose $\Delta = 0$ over the FM wire although we allow the SC field to be induced in the FM wire by proximity. For this heterostructure we utilise a 2D lattice model described by the following simple Hamiltonian,

$$\mathcal{H} = \sum_{\boldsymbol{i},\boldsymbol{j},s}(tf_{\boldsymbol{i},\boldsymbol{j}} + J^x_{\boldsymbol{i}}g^x_{\boldsymbol{i},\boldsymbol{j}} + J^y_{\boldsymbol{i}}g^y_{\boldsymbol{i},\boldsymbol{j}})\psi^\dagger_{\boldsymbol{i},s}\psi_{\boldsymbol{j},s} + \sum_{\boldsymbol{i}}\mu_{\boldsymbol{i}}\cdot n_{\boldsymbol{i}} - \sum_{\boldsymbol{i}}\psi^\dagger_{\boldsymbol{i},s}(\boldsymbol{h}_{\boldsymbol{i}}\cdot\boldsymbol{\sigma})_{ss'}\psi_{\boldsymbol{i},s'} + \sum_{\boldsymbol{i}}(\Delta_{\boldsymbol{i}}\psi^\dagger_{\boldsymbol{i},\uparrow}\psi^\dagger_{\boldsymbol{i},\downarrow} + h.c.)\,, \quad \text{(S9)}$$

where $\psi_{\boldsymbol{i},s}$ is the annihilation operator of fermions at lattice site $\boldsymbol{i}$ with spin s, $n_{\boldsymbol{i}}$ is the local charge density operator, while μ and $\boldsymbol{h}$ stands for the chemical potential and Zeeman field respectively. With t we denote the transfer integral associated with the even in inversion connection matrix $f_{\boldsymbol{i},\boldsymbol{j}} = \delta_{\boldsymbol{j},\boldsymbol{i}\pm\boldsymbol{x}} + \delta_{\boldsymbol{j},\boldsymbol{i}\pm\boldsymbol{y}}$, where $\boldsymbol{x}$ and $\boldsymbol{y}$ the unit vectors along the x- and y- coordinate axes, corresponding to nearest neighbours lattice points. On the other hand for the charge current J^x and J^y we associate the odd in inversion connection matrices $g^x_{\boldsymbol{i},\boldsymbol{j}} = \pm i\delta_{\boldsymbol{j},\boldsymbol{i}\pm\boldsymbol{x}}$ and $g^y_{\boldsymbol{i},\boldsymbol{j}} = \pm i\delta_{\boldsymbol{j},\boldsymbol{i}\pm\boldsymbol{y}}$ respectively. Hamiltonian Eq. (S9) acquires the following compact form when we introduce the spinor $\Psi^\dagger_{\boldsymbol{i}} = \left(\psi^\dagger_{\boldsymbol{i},\uparrow}, \psi^\dagger_{\boldsymbol{i},\downarrow}, \psi_{\boldsymbol{i},\uparrow}, \psi_{\boldsymbol{i},\downarrow}\right)$ and employ the usual Pauli matrices $\boldsymbol{\tau}$, $\boldsymbol{\sigma}$ acting on the Nambu and spin space respectively.

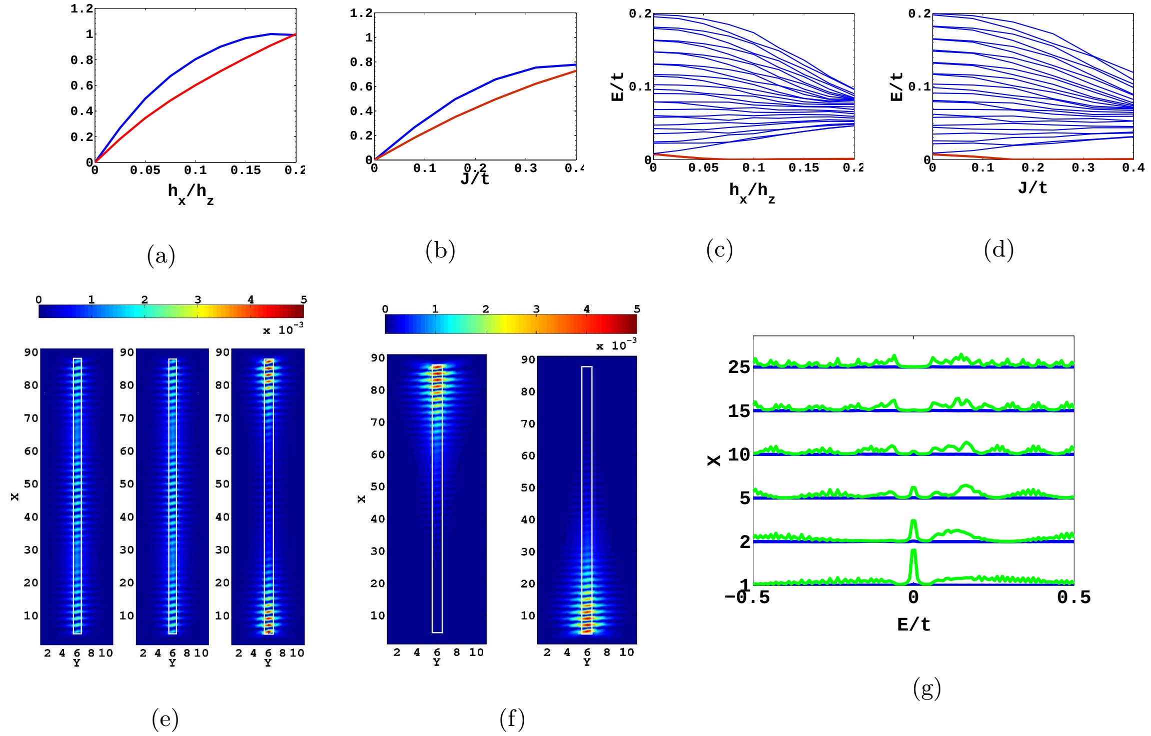

FIG. S3: 1D FM wire with length L=83 lattice sites on a SC with dimensions 91×11. For the SC region we consider $\Delta = 1$ and $\mu_{SC} = 0$, while for the FM wire $h_z = 4$ and $\mu_{FM} = 3.8$ (all in t units) corresponding to a single spin band occupied. In the middle of the FM wire the induced equal spin SC P_y (blue line) and SO A_y correlations (red line) a) for $J = 0.08$ as the Zeeman field h_y increases and b) for $h_y = 0.025h_z$ for increasing supercurrent flow J. The correlations are normalised with respect to the maximum value of P_y correlations. For $J = 0.08$ c) and $h_y = 0.025h_z$ d) the energy gap in the FM wire created due to the induced p_y SC field as the transverse Zeeman field h_y and the supercurernt flow J increase respectively. Because for $h_z = 4$ and $\mu_{FM} = 3.8$ the FM wire is in a non-trivial topolocigal phase the lowest energy (red line) corresponding to a MF tends to zero. e) The lowest energy $|\Psi|^2$ wavefunction for $h_z = 4$ and for $h_y = 0.05h_z$, $|J| = 0$ (right), $h_y = 0$, $|J| = 0.08$ (middle), $h_y = 0.05h_z$, $|J| = 0.08$ (left). Only when both h_y and J fields are finite, a localised eigenstate emerges at the edges of the FM wire. f) The MFs wavefunctions for $h_y = 0.05h_z$, $|J| = 0.08$. Experimentally the MFs wavefunctions can by identified by zero-bias conductance maps based on scanning tunneling microscopy measurement. g) The local density of states along the FM wire for $h_y = 0.4$, $|J| = 0.2$. A peak at E=0 appears only at the edge of the FM wire (x=1-5), while an energy gap $\Delta E \simeq 0.05$ emerges over the middle area of the FM wire. We remark that the local density of states presented here, is a possible experimental signature for the emergence of zero energy MFs in scanning tunneling microscopy experiments.

$$\mathcal{H} = \frac{1}{2}\sum_{\boldsymbol{i},\boldsymbol{j}} \Psi^\dagger_{\boldsymbol{i}} \hat{H}_{\boldsymbol{i},\boldsymbol{j}} \Psi_{\boldsymbol{j}}, \quad \hat{H}_{\boldsymbol{i},\boldsymbol{j}} = t f_{\boldsymbol{i},\boldsymbol{j}}\tau_3 + J^x_{\boldsymbol{i}} g^x_{\boldsymbol{i},\boldsymbol{j}} + J^y_{\boldsymbol{i}} g^y_{\boldsymbol{i},\boldsymbol{j}} + (\mu_{\boldsymbol{i}}\tau_3 - \tau_3 \boldsymbol{h}_{\boldsymbol{i}} \cdot \tilde{\boldsymbol{\sigma}} + \Delta_{\boldsymbol{i}}\tau_2\sigma_2)\delta_{\boldsymbol{i},\boldsymbol{j}}\,, \tag{S10}$$

where $\tilde{\boldsymbol{\sigma}} = (\sigma_1, \tau_3\sigma_2, \sigma_3)$. We diagonalise the above Hamiltonian by solving the Bogoliubov-de Gennes (BdG) equation $\sum_{\boldsymbol{j}} \hat{H}_{\boldsymbol{i},\boldsymbol{j}} U_{\boldsymbol{j},n} = E_n U_{\boldsymbol{i},n}$, where E_n and $U_{\boldsymbol{i},n} = \left(u^*_{\boldsymbol{i},\uparrow}, u^*_{\boldsymbol{i},\downarrow}, v_{\boldsymbol{i},\uparrow}, v_{\boldsymbol{i},\downarrow}\right)^T$ the energy eigenvalues and the corresponding eigenfunctions respectively. Using the eigenvalues and eigenstates of the system we can calculate any correlations at lattice site $\boldsymbol{i}$ corresponding to the representation $\hat{O}$ in the SU(4) spin-Nambu space based on the following equation

$$\sum_{\boldsymbol{j}} \left\langle \Psi^\dagger_{\boldsymbol{i}} \hat{O} f^r_{\boldsymbol{i},\boldsymbol{j}} \Psi_{\boldsymbol{j}} \right\rangle = \sum_{n,\boldsymbol{j}} [U^\dagger_{n,\boldsymbol{i}} \hat{O} f^r_{\boldsymbol{i},\boldsymbol{j}} U_{\boldsymbol{j},n}] n_F(E_n)\,, \tag{S11}$$

where $n_F(E_n) = \frac{1}{1+e^{\beta E_n}}$ the Fermi distribution and the connection matrix $f^r_{\boldsymbol{i},\boldsymbol{j}}$, a basis function of the C_{4u} point group symmetry which corresponds to the coordinate space configuration of the particular correlations.

Due to the coupling of the J, h_y and Δ fields, an equal spin pairing field of the form $\Delta^p_y g^x_{\boldsymbol{i},\boldsymbol{j}}\tau_2$ emerges (Quartet B). In the same way J and h_y couple to the chemical potential of the SC or to an effective chemical potential present in the boundaries of the FM/SC heterostructure due to a Fermi mismatch of the two systems, to induce SO interactions of the form $\alpha_y g^x_{\boldsymbol{i},\boldsymbol{j}}\tau_3\sigma_2$ (Quartet A). These fields which emerge on the boundaries of the FM/SC heterostructure mediate by proximity within the FM wire.

In Fig. S3 we present typical results obtained by diagonalising Eq. S10 for a 91×11 SC with a 1D FM wire with length $L = 83$ located at the center of the system, considering $\Delta = 1$, $\mu_{SC} = 0$ for the SC and $h_z = 4$, $\mu_{FM} = 3.8$ (all in t units) for the FM wire. For these parameters the FM wire is strongly polarised with a single spin band occupied. Notice also that due to their antiparallel with respect to the FM wire configuration the supercurrent and the transverse Zeeman field vanish along the wire. We demonstrate (Fig. S3 a) and b)) how equal spin SC correlations Δ^p_y and SO correlations A_y emerge within the wire as the supercurrent flow J and the Zeeman field h_y acquire finite values. Notice that for $J = 0$ *or* $h_y = 0$ there are no induced triplet correlations Δ^p_y and A_y and therefore a topological non-trivial SC phase cannot be realised. In Fig. S3 c) and d) we present how the energy gap and a single zero-energy excitation emerges with increasing h_y and J respectively due to the induced p SC fields. The zero-energy excitation localised at the edges of the FM wire emerges only when *both* current J and Zeeman field h_y are finite Fig. S3 e). Particularly for $|h_y| = 0.1$ and $|J| = 0.2$ the local density of states of the FM wire (Fig. S3 g)) reveals the energy gap in the quasiparticle excitation spectrum of the FM wire due to the induced p SC field. For the same parameters a pair of zero-energy bound states emerges localised at the edges of the wire (Fig. S3 f)) . The zero energy Majorana bound states derive by expressing the two zero-energy eigenstates of Hamiltonian Eq. S10 in the Majorana basis $\Gamma = \left(\gamma_{\boldsymbol{i},\uparrow}, \gamma_{\boldsymbol{i},\downarrow}, \overline{\gamma}_{\boldsymbol{i},\uparrow}, \overline{\gamma}_{\boldsymbol{i},\downarrow}\right)^T$, where $\gamma_{\boldsymbol{i},s} = \gamma^\dagger_{\boldsymbol{i},s}$ and $\overline{\gamma}_{\boldsymbol{i},s} = \overline{\gamma}^\dagger_{\boldsymbol{i},s}$ the Majorana operators. The Majorana basis relates to the Nambu spin basis in the following way $\Gamma = A\Psi$ where $A = e^{i\frac{\pi}{4}\tau_2} e^{-i\frac{\pi}{4}\tau_1} e^{i\frac{\pi}{4}}$.

The following 1D effective Hamiltonian for the FM wire including the induced fields a_y and Δ^p_y, gives a better understanding for the emergence of MFs in the particular FM/SC heterostructure.

$$H^{1D,eff}_{FM} = \frac{1}{2}\sum_i \Psi^\dagger_i \left[t' f^x_{i,j}\tau_3 + (\mu_{FM}\tau_3 - h_z\tau_3\sigma_3 + \Delta'\tau_2\sigma_2)\delta_{i,j} + \left(\alpha_y\tau_3\sigma_2 + \Delta^p_y\tau_2 + \Im\Delta^p_x\tau_2\sigma_3\right) g^x_{i,j}\right] \Psi_j \,, \quad \text{(S12)}$$

We have also included the induced by proximity conventional SC field Δ' and the $\Im\Delta^p_x g^x_{i,j}\tau_2\sigma_3$ SC field induced by the coupling of chemical potential μ_{FM} and Zeeman field h_z with Δ^p_y(see Sec. I). Moreover we take into account a possible renormalisation of the dispersion of the FM wire $t' \leq t$ due to its coupling with the SC [3]. As elaborated in Sec. III Hamiltonian Eq. S12 belongs to the chiral BDI symmetry class with a strong integer $\mathbb{Z}$ topological invariant. The criteria for realising the topologically non-trivial phase with topological invariant $\mathcal{W} = 1$ are $|2t' - \sqrt{h_z^2 - \Delta'^2}| < |\mu_{FM}| < |2t' + \sqrt{h_z^2 - \Delta'^2}|$ corresponding to a single energy band of the FM wire occupied. The chemical potential of the FM wire for the particular case presented above is within the boundaries set by the topological criteria. Thus, the emergence of the zero energy MFs at the edges of the FM wire is completely justified.

b. Quasi-1D FM wire Next we elaborate the case of a quasi-1D FM wire with finite width. In this case we have also to take into account the induced SOC of the form $\alpha_x g^y_{\boldsymbol{i},\boldsymbol{j}}\sigma_1$ due to the rotation of the Zeeman field around the x-axis [4]. As presented in Sec. I these spin-orbit interactions couple to the singlet SC field Δ, the Zeeman field h_z and the chemical potential μ_{FM} of the wire, to induce triplet p SC fields of the form $\Im\Delta^p_y g^y_{\boldsymbol{i},\boldsymbol{j}}\tau_1$ and $\Delta^p_x g^y_{\boldsymbol{i},\boldsymbol{j}}\tau_1\sigma_3$. In Fig. S4 e) and f) we present the induced fields on the W=3 FM wire embedded in a conventional SC derived by diagonalising Eq. S10 considering $\Delta = 2$ (only within the SC region while $\Delta = 0$ within the wire), $\mu_{SC} = 0$, $h_z = 4$, $|h_y| = 1.6$ and $|J| = 0.2$. From Fig. S4 b) we observe that zero-energy modes (which acquire a small finite energy due to finite length of the wire) emerge for $0.9 < \mu_{FM} < 2.4$ (see Fig. S4 c) and $5.8 < \mu_{FM} < 7.2$ when one transverse sub-band is occupied and for $3.6 < \mu_{FM} < 4.5$ when 3 transverse sub-bands are occupied. For $3.6 < \mu_{FM} < 4.5$ apart from the zero-energy localised modes (red line) two gapped states corresponding to complex fermions also localised at the wire's edges emerge (green,purple lines). Finally in Fig. S4 d) we present the lowest (almost zero)-energy eigenstate for $\mu = 0.95h_z$ in the Majorana basis. Again we interpret our results according to the following effective Hamiltonian for the quasi-1d FM wire, which includes the induced fields.

$$\begin{aligned} H^{Q1D,eff}_{FM} = \frac{1}{2}\sum_{\boldsymbol{i},\boldsymbol{j}} \Psi^\dagger_{\boldsymbol{i}} \Big[&t' f_{\boldsymbol{i},\boldsymbol{j}}\tau_3 + (\mu_{FM}\tau_3 - h_z\tau_3\sigma_3 - h_y\sigma_2 + \Delta'\tau_2\sigma_2)\delta_{\boldsymbol{i},\boldsymbol{j}} \\ &+ (\alpha_y\tau_3\sigma_2 + \Delta^p_y\tau_2 + \Im\Delta^p_x\tau_2\sigma_3 + J^x) g^x_{\boldsymbol{i},\boldsymbol{j}} + (\alpha_x\sigma_1 + \Delta^p_x\tau_1\sigma_3 + \Im\Delta^p_y\tau_1 + J^y) g^y_{\boldsymbol{i},\boldsymbol{j}}\Big] \Psi_{\boldsymbol{j}} \,, \end{aligned} \quad \text{(S13)}$$

We have also included small components of the supercurrent flow J which are induced in the FM wire by proximity. For simplicity we ignore in our analysis the dependence of the fields in the transverse direction. Moreover as explained in the main article the in-plane Zeeman field h_y in this case can also emerge from a rotation of the magnetisation of the FM wire (see Fig. S4 a)). Therefore we have also included the corresponding term $h_y\sigma_2$ in the effective Hamiltonian Eq. S13. As described in Sec. III the SOC α_x and the Zeeman field h_y as well as the induced triplet SC fields Δ^p_x and $\Im\Delta^p_y$ don 't satisfy the condition $\boldsymbol{d}_{\Im p} = \boldsymbol{d}_\alpha \times \boldsymbol{d}_h = \boldsymbol{d}_p \times \boldsymbol{d}_h$ and therefore break the chiral symmetry.

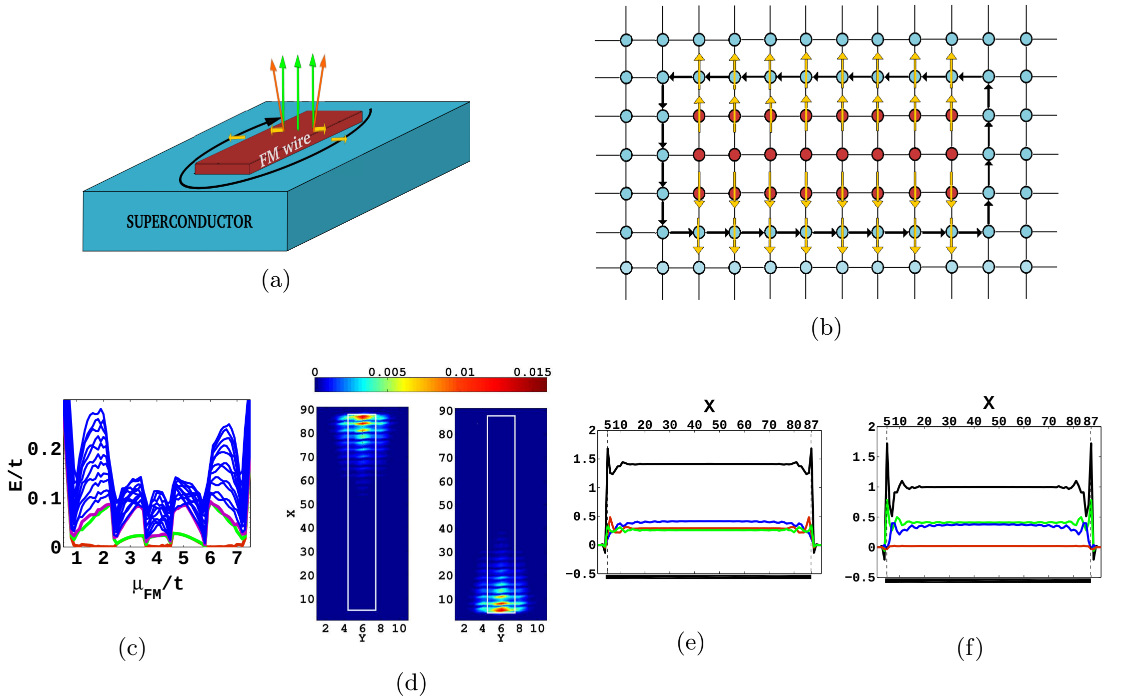

FIG. S4: a) A quasi-1D FM wire embedded in conventional superconductor. The main component of the wire's magnetization is perpendicular to SC surface (green arrows). The magnetic field created by wire magnetization stimulates a supercurrent flow around the wire (black arrow). A rotation of the magnetization of the wire contributes a Zeeman field h_y (yellow arrows) which is not screened by the super-current and leaks into the SC region. b) Schematic 2D lattice modelization of the three rows wide wire (red sites) embedded in a SC (blue sites) corresponding to the Hamiltonian (Eq. 1) in the Letter. c) For a three rows wide FM wire with length L=83, the low-energy excitation spectrum as the chemical potential of the FM wire increases, taking $\Delta = 2$, $\mu_{SC} = 0$, $|h_y| = 1.6$, $|J| = 0.2$ and $h_z = 4$. A single zero-energy excitation emerges (red line) when an odd number of bands of the wire cross the Fermi level. d) For $\mu_{FM} = 2t$ the two Majorana modes localized at the edges of the wire. e) and f) The induced on the FM wire correlations conjugate to the triplet SC field $\Delta^p_y g^x_{\boldsymbol{i},\boldsymbol{j}} \tau_2$ (blue line) and SOC $\alpha_y g^x_{\boldsymbol{i},\boldsymbol{j}} \tau_3 \sigma_2$ (red line) along the x-axis and the same for the triplet SC field $\Im \Delta^p_y g^y_{\boldsymbol{i},\boldsymbol{j}} \tau_1$ (black line) and SOC $\alpha_x g^y_{\boldsymbol{i},\boldsymbol{j}} \sigma_1$ (green line) along the y-axis, for the edge rows and the middle row respectively. The overall induced SOC $\alpha_y g^x_{\boldsymbol{i},\boldsymbol{j}} + \alpha_x g^y_{\boldsymbol{i},\boldsymbol{j}}$ is of the Rashba form while $\Delta^p_y g^x_{\boldsymbol{i},\boldsymbol{j}} + \Im \Delta^p_y g^y_{\boldsymbol{i},\boldsymbol{j}}$ corresponds to a chiral SC field. All values are normalized with respect to the value of the correlations for the $\Im \Delta^p_y g^y_{\boldsymbol{i},\boldsymbol{j}} \tau_1$ field in the middle of the wire.

Since the time reversal symmetry is also broken the system described by the Hamiltonian Eq. S13 belongs to the D symmetry class which accepts a $\mathbb{Z}_2$ topological invariant (1D systems). Quasi-1D systems described by Hamiltonian S13 without the current J and h_y terms, were investigated in Ref. [5]. There it was found that when the width W of the system is smaller than the pairing coherence length $\xi_0 \sim t/\Delta^p$ where Δ^p the single electron species pairing field, a single pair of Majorana zero energy modes localised at the edges of the wire emerge when odd number of transverse sub-bands are occupied. We obtain the same results in our model where the p-wave pairing field is induced due to the supercurrent flow J and the transverse Zeeman field h_y, even though the latter fields acquire a small but finite value within the wire. According to our results in this case $\frac{1}{\xi} \sim \Delta^p/t < 0.1$ and the corresponding coherence length $\xi > W$ suffice for Majorana zero-energy modes to emerge localised at the edge of the wire. Moreover from the topological phase diagram (Fig. S4 c)) we conclude that the transverse sub-bands of the quasi-1D wire are slightly renormalised due to the coupling with the SC [3] and particularly 2 sub-bands acquire a bandwidth which corresponds to $t' \simeq 0.9$ while for the third sub-band we get $t' \simeq 0.85$. Regarding the case for the quasi-1D wire coupled to a SC with $\Delta = 1$ presented in the main article, from the corresponding topological phase diagram (Fig. 4) we derive the renormalised kinetic terms $t' \simeq 0.875$ (for two sub-bands) and $t' \simeq 0.775$ (for one sub-band). In general we conclude that the renormalisation is not the same for all the transverse sub-bands, while as anticipated, it increases as the singlet pairing field Δ of the substrate SC decreases.

c. Superconductor-Ferromagnet-Superconductor trilayers Finally we present the induced SO interactions and the p pairing field for the SC/FM/SC trilayers case with [001] interfaces. The microscopic Hamiltonian in this case acquires the following form

$$\mathcal{H} = \sum_{\boldsymbol{i},\boldsymbol{j},l,s} (tf_{\boldsymbol{i},\boldsymbol{j}} + \boldsymbol{J}\boldsymbol{g}_{\boldsymbol{i},\boldsymbol{j}})\psi^\dagger_{\boldsymbol{i},l,s}\psi_{\boldsymbol{j},l,s}$$
$$- \sum_{\boldsymbol{i},l}\left[\mu_l \cdot n_{\boldsymbol{i},l} - \psi^\dagger_{\boldsymbol{i},l,s}(\boldsymbol{h}_l\cdot\boldsymbol{\sigma})_{ss'}\psi_{\boldsymbol{i},l,s'} + \Delta_l\psi^\dagger_{\boldsymbol{i},l,\uparrow}\psi^\dagger_{\boldsymbol{i},l,\downarrow} + \Delta_l\psi_{\boldsymbol{i},l,\downarrow}\psi_{\boldsymbol{i},l,\uparrow}\right] + \sum_{\boldsymbol{i},l,l',s} t_{l,l'}\psi^\dagger_{\boldsymbol{i},l,s}\psi_{\boldsymbol{i},l',s} + c.c.\,, \quad \text{(S14)}$$

where l is the layer index. In addition to Eq. S9 we have considered the simplest coupling term between the layers $t_{l,l'}\psi^\dagger_{\boldsymbol{i},l,s}\psi_{\boldsymbol{i},l',s}$, while we have dropped the lattice site index $\boldsymbol{i}$ from the fields $\boldsymbol{J}$,μ ,$\boldsymbol{h}$ and Δ as they are considered to be uniform within each layer. Introducing the spinor $\Psi^\dagger_{\boldsymbol{i},l} = \left(\psi^\dagger_{\boldsymbol{i},l,\uparrow}, \psi^\dagger_{\boldsymbol{i},l,\downarrow}, \psi_{\boldsymbol{i},l,\uparrow}, \psi_{\boldsymbol{i},l,\downarrow}\right)$ for each layer and the Pauli matrices $\boldsymbol{\tau}$, $\boldsymbol{\sigma}$, Hamiltonian Eq. S15 simplifies to

$$\mathcal{H} = \frac{1}{2}\sum_{\boldsymbol{i},\boldsymbol{j},l}\Psi^\dagger_{\boldsymbol{i},l}\hat{H}_{\boldsymbol{i},\boldsymbol{j},l}\Psi_{\boldsymbol{j},l} + \frac{1}{2}\sum_{\boldsymbol{i},l,l'}\Psi^\dagger_{\boldsymbol{i},l}\hat{H}_{\boldsymbol{i},l,l'}\Psi_{\boldsymbol{i},l'},$$
$$\hat{H}_{\boldsymbol{i},\boldsymbol{j},l} = t_l f_{\boldsymbol{i},\boldsymbol{j}}\tau_3 + J_l g_{\boldsymbol{i},\boldsymbol{j}} + (\mu_l\tau_3 - \tau_3\boldsymbol{h}_l\cdot\tilde{\boldsymbol{\sigma}} + \Delta_l\tau_2\sigma_2)\delta_{\boldsymbol{i},\boldsymbol{j}}, \qquad \hat{H}_{\boldsymbol{i},l,l'} = t_{l,l'}\tau_3\,, \quad \text{(S15)}$$

Particularly we consider a finite singlet pairing field $\Delta \neq 0$ only for the two superconducting layers and a Zeeman field $h_z\tau_3\sigma_3 \neq 0$ within the ferromagnetic layer only. Moreover a supercurrent flow $J^x_l g^x_{\boldsymbol{i},\boldsymbol{j}}$ along the x-axis and a Zeeman field $h_y\sigma_2$ are externally applied to the two superconducting layers in an antiparallel configuration as depicted in Fig. S5. For the coupling term $t_{l,l'}\tau_3$ we consider a finite value only between the ferromagnetic and the superconducting layers, while the two superconducting layers are not coupled with each other. As elaborated Appendix I the current $J^x g^x_{\boldsymbol{i},\boldsymbol{j}}$ and the Zeeman field $h_y\sigma_2$ couple to the singlet pairing field $\Delta\tau_2\sigma_2$ and the chemical potential $\mu\tau_3$ to induce a triplet pairing field $\Delta^p_y\tau_2 g^x_{\boldsymbol{i},\boldsymbol{j}}$ and SOC $a_y\tau_3\sigma_2 g^x_{\boldsymbol{i},\boldsymbol{j}}$ respectively. The pairing field Δ^p_y and the SOC a_y are subsequently induced by proximity to the ferromagnetic layer (see Fig. S5). Finally the pairing field $\Delta^p_y\tau_2 g^x_{\boldsymbol{i},\boldsymbol{j}}$ couples to Zeeman field $h_z\tau_3\sigma_3$ and the chemical potential $\mu\tau_3$ of the ferromagnetic layer to induce an additional triplet pairing field $\Im\Delta^p_x\tau_2\sigma_3 g^x_{\boldsymbol{i},\boldsymbol{j}}$. In Fig. S5 we present the results derived from Eq. S15 for a trilayer with $\Delta = 2$, $\mu_{SC} = 0$, $|J^x| = 0.2$, $|h_y| = 0.8$ for the SC layers and $h_z = 8$, $\mu_{FM} = 6.4$ for the FM layer. Notice the corresponding correlations for the induced fields Δ^p_y and a_y in Fig. S5 b) and c) respectively.

In this case the effective Hamiltonian of the ferromagnetic layer including the induced fields acquires the following form.

$$H^{2D,eff}_{FM} = \frac{1}{2}\sum_{\boldsymbol{i},\boldsymbol{j}}\Psi^\dagger_{\boldsymbol{i}}\left[t' f_{\boldsymbol{i},\boldsymbol{j}}\tau_3 + (\mu_{FM}\tau_3 + h_z\tau_3\sigma_3 + \Delta'\tau_2\sigma_2)\delta_{\boldsymbol{i},\boldsymbol{j}} + (\alpha_y\tau_3\sigma_2 + \Delta^p_y\tau_2 + \Im\Delta^p_x\tau_2\sigma_3)g^x_{\boldsymbol{i},\boldsymbol{j}}\right]\Psi_{\boldsymbol{j}}\,, \quad \text{(S16)}$$

where we consider $t' \leq t$ as the renormalised kinetic term within the ferromagnetic layer due to coupling with the superconducting layers. Because all the induced fields follow the direction of the applied current flow the only transverse term in Eq.S13 is the kinetic term $t' f_{\boldsymbol{i},\boldsymbol{j}}\tau_3$. Thus we can rewrite the above Hamiltonian in the following form

$$H^{2D,eff}_{FM} = \frac{1}{2}\sum_{i,j,\nu,\nu'}\Psi^\dagger_{i,\nu}\left[t' f_{i,j}\tau_3 + (\mu_{FM}\tau_3 + h_z\tau_3\sigma_3 + \Delta'\tau_2\sigma_2)\delta_{i,j}\right.$$
$$\left. + \ (\alpha_y\tau_3\sigma_2 + \Delta^p_y\tau_2 + \Im\Delta^p_x\tau_2\sigma_3)g^x_{i,j} + t'_\perp\delta_{\nu,\nu\pm1}\tau_3\right]\Psi_{j,\nu'}$$
$$H^{2D,eff}_{FM} = \frac{1}{2}\sum_{i,\nu}\Psi^\dagger_{i,\nu}\left[H^{1D}_{i,j,\nu} + t'_\perp\delta_{\nu,\nu\pm1}\tau_3\right]\Psi_{j,\nu'}\,, \quad \text{(S17)}$$

where $\nu \in (1, 2, ..., N_y)$ indicates each row of the ferromagnetic layer for which we have introduced the spinor $\Psi^\dagger_{i,\nu} = \left(\psi^\dagger_{i,\nu,\uparrow}, \psi^\dagger_{i,\nu,\downarrow}, \psi_{i,\nu,\uparrow}, \psi_{i,\nu,\downarrow}\right)$. The 1D Hamiltonian $H^{1D}_{i,j,\nu}$ for each row has exactly the same form as Eq. S12. First we assume periodic boundary conditions along the transverse direction. Because the fields in Eq. S15 are uniform we can drop the row index from the 1D effective Hamiltonians, $H^{1D}_{i,j,\nu} = H$. Since the system acquires the translation symmetry across the transverse direction we can block diagonalise Hamiltonian Eq. S17 by conducting a Fourier transformation from y-coordinate to k_y-momenta space. After the transformation the effective Hamiltonian of the ferromagnetic layer acquires the following form $H^{eff}_{FM} = \frac{1}{2}\sum_{i,k_y}\Psi^\dagger_{i,k_y}\left[H^{1D}_{i,j} + t'_\perp\tau_3\lambda_{k_y}\right]\Psi_{j,k_y}$, where $\lambda_{k_y} = 2\cos(k_y)$ the eigenvalues of the transverse Hamiltonian $H_\perp = \delta_{\nu,\nu\pm1}$ with $k_y = \frac{2\pi(n-1)}{N_Y}$ and $n \in (1, 2, ..., N_y)$. For open boundary conditions the system maintains only the reflection symmetry along the y-axis direction and therefore the 1D Hamiltonians $H^{1D}_{i,j,\nu}$ in general can be related in the following way $H^{1D}_{i,j,m+1} = H^{1D}_{i,j,N_y-m}$ where $m \in [0, (N_y-2)/2]$ for N_y even and $m \in [0, (N_y-3)/2]$ for N_y odd. In the eigenbasis of the reflection symmetry matrix R Hamiltonian

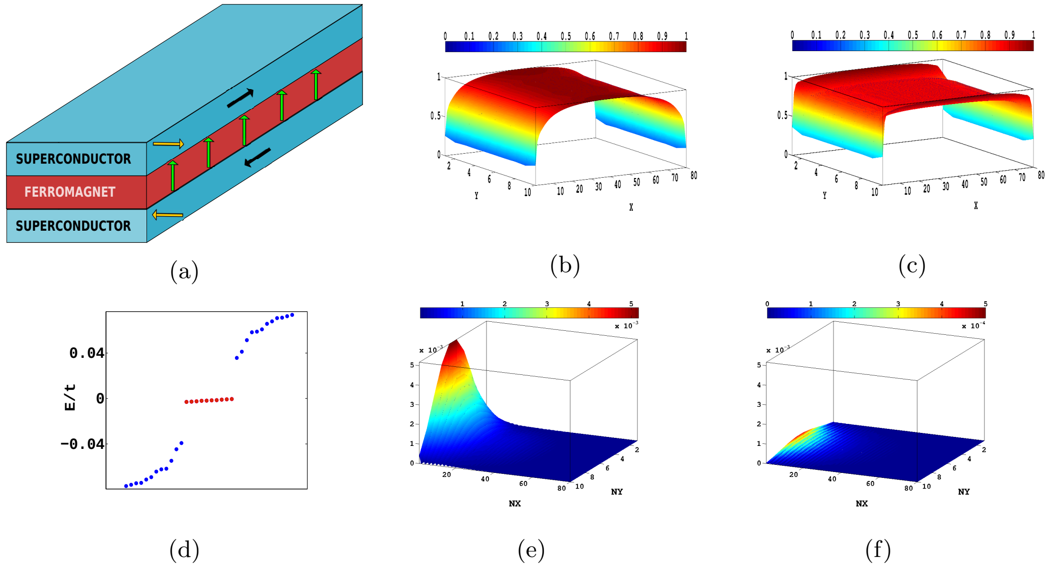

FIG. S5: a) A superconductor-ferromagnet-superconductor trilayer, where supercurrent flows (black arrows) and Zeeman fields (yellow arrows), perpendicular to the magnetisation of the FM (green arrows), are externally applied in an antiparallel configuration on the SC layers. The induced triplet pairing $P_y\tau_2 g^x_{\boldsymbol{i},\boldsymbol{j}}$ b) and SO $A_y\tau_3\sigma_2 g^x_{\boldsymbol{i},\boldsymbol{j}}$ c) correlations within the FM layer with $h_z = 8$ and $\mu_{FM} = 6.4$ coupled to two SC layers with $\Delta = 2$, $|h_y| = 0.8$, $|J^x| = 0.4$ and $t_c = 0.8$ for the interlayer hopping term. The induced correlations are normalised to their maximum value. d) For the same parameters the low-energy excitation spectrum. Notice with red color the 5 pairs of almost zero energy eigenstates separated by the rest excitations by an energy gap $\Delta E \simeq 0.04$. For $\mu = 6.4$ the topological invariant is indeed $\mathcal{W} = 5$ (see Fig. 4b in main article). The left edge localised Majorana wavefunction corresponding to eigenenergy $E = 2.5 \cdot 10^{-4}$ within the FM layer e) and the two SC layers f). The Majorana wavefunction corresponds to spin down within the FM layer and to spin up within the SC.

Eq. S17 acquires a block diagonal form $H^{eff}_{FM} = \frac{1}{2}\sum_{i,n} \Psi^\dagger_{i,n}\left[H^{1D}_{i,j,n} + t'_\perp\tau_3\lambda_n\right]\Psi_{j,n'}$ [6], with λ_n the eigenvalues of the transverse part of the Hamiltonian $H_\perp = \delta_{\nu,\nu\pm1}$ where in this case terms connecting the first and the last row are absent. In both cases notice that the kinetic term along the transverse direction acts as an effective chemical potential for each of the N_y independent subsystems described by the Hamiltonian $H^{1D}_{i,j,n}$ which belongs to the BDI chiral symmetry class. Thus the overall system can support $\mathcal{W} = \sum_n \mathcal{W}_n$ pairs of zero-energy Majorana modes, where $\mathcal{W}_n$ is the integer topological invariant for each subsystem $H^{1D}_{i,j,n} + t'_\perp\tau_3\lambda_n$. Due to the effective chemical potential $t'_\perp\lambda_n$ the topological criteria are modified accordingly and particularly for realising a phase with topological invariant $|\mathcal{W}_n| = 1$ we get $|2t' - \sqrt{h_z^2 - \Delta^2}| < |\mu + t'_\perp\lambda_n| < |2t' + \sqrt{h_z^2 - \Delta^2}|$ (see Sec. III). For closed boundary conditions we observe that apart from $k_y = 0$ (and in addition $k_y = \pi$ when N_y is even) eigenvalues λ_n are doubly degenerate. Moreover when N_y is even the eigenvalues are symmetric with respect to 0. Therefore when $\sqrt{h_z^2 - \Delta^2} > 2t'$, for N_y even the topological invariant $\mathcal{W}$ is odd apart from $|\mu - \sqrt{h_z^2 - \Delta^2}| < 2|(t' - t'_\perp)|$, while for N_y odd the topological invariant is odd only when $\mu > 2(t'_\perp - t') + \sqrt{h_z^2 - \Delta^2}$. For open boundary conditions the eigenvalues λ_n are always non-degenerate and therefore transitions among topological phases with odd and even topological invariant are in general observed. The symmetry of the eigenvalues with respect to zero is reflected in the symmetry of the topological phases with respect to $\sqrt{h_z^2 - \Delta^2}$ in Fig. 4b of the main article.

d. Results for self-consistently determined pairing field Δ. Finally in Fig. S6 we present typical results for the 1D and quasi-1D wire on a conventional SC where the singlet pairing field Δ of the superconductor is determined self-consistently considering an attractive on-site potential U. Including the on-site interaction term Hamiltonian Eq. S9 modifies to

$$\mathcal{H} = \sum_{\boldsymbol{i},\boldsymbol{j},s}(tf_{\boldsymbol{i},\boldsymbol{j}} + J^x_{\boldsymbol{i}} g^x_{\boldsymbol{i},\boldsymbol{j}} + J^y_{\boldsymbol{i}} g^y_{\boldsymbol{i},\boldsymbol{j}})\psi^\dagger_{\boldsymbol{i},s}\psi_{\boldsymbol{j},s} + \sum_{\boldsymbol{i}} \mu_{\boldsymbol{i}} \cdot n_{\boldsymbol{i}} - \sum_{\boldsymbol{i}} \psi^\dagger_{\boldsymbol{i},s}(\boldsymbol{h_i} \cdot \boldsymbol{\sigma})_{ss'}\psi_{\boldsymbol{i},s'} + \sum_{\boldsymbol{i}} U_{\boldsymbol{i}}\psi_{\boldsymbol{i},\downarrow}\psi_{\boldsymbol{i},\uparrow}\psi^\dagger_{\boldsymbol{i},\uparrow}\psi^\dagger_{\boldsymbol{i},\downarrow}\,. \quad \text{(S18)}$$

From the above Hamiltonian for interacting fermions we retrieve the corresponding non-interacting fermions Hamiltonian Eq. S9 using the mean field approach for the singlet pairing field

$$\Delta_{\boldsymbol{i}} = U_{\boldsymbol{i}} < \psi_{\boldsymbol{i},\downarrow}\psi_{\boldsymbol{i},\uparrow} >= \frac{1}{2}U_{\boldsymbol{i}}\sum_{\boldsymbol{j}}\left\langle \Psi^\dagger_{\boldsymbol{i}}[\tau_2\sigma_2\delta_{\boldsymbol{i},\boldsymbol{j}}]\Psi_{\boldsymbol{j}}\right\rangle\,, \quad \text{(S19)}$$

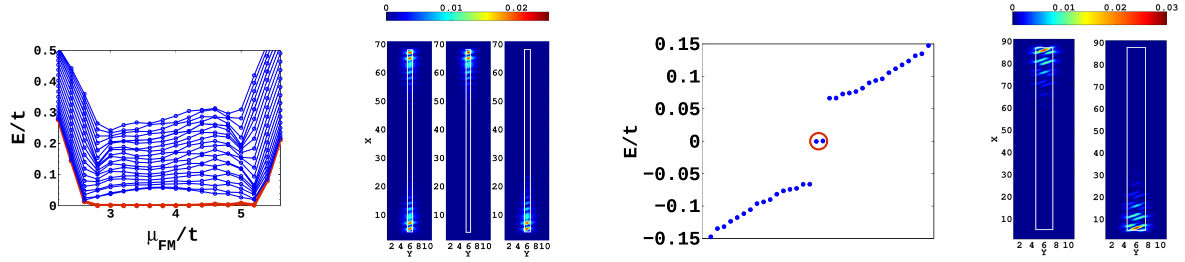

(a) (b) (c) (d)

FIG. S6: a) The low-energy excitation spectrum for a 1D FM wire with length L=63 lattice sites on a conventional SC region with dimensions 71×11. The SC field was determined *self-consistently* considering an on-site potential U=1 only within the SC region and $\mu_{SC} = 0$, $h_z = 4$, $|h_y| = 0.4$ and $|J| = 0.2$. The lowest eigenergy (red line) approaches to zero for $h_z - 1.2 < \mu_{FM} < h_z + 1.2$ as anticipated by the topological criterion Eq. S28 corresponding to a remormalised dispersion $t' = 0.6t$. b) left) The lowest energy wavefunction $|\Psi|^2$ for $\mu_{FM} = 4$ and right) one of the corresponding Majorana fermions localised at one edge of the FM wire. c) The excitation energy spectrum for a 3 rows wide FM wire coupled to a conventional SC. The SC field was determined *self-consistently* for on-site potential U=2 and $\mu_{SC} = 0$ $|h_y| = 0.8$, $|J| = 0.2$ and , $h_z = \mu_{FM} = 6$. d) The near zero energy eigenstates (within red circle) separated by a $\Delta E \simeq 0.07$ energy gap from the rest excitation spectrum, correspond to the two Majorana fermions localised at the edges of the wire.

Particularly Fig. S6 a) is the topological phase diagram with respect to the chemical potential μ_{FM} of the 1D FM wire considering $U = 1$ only within the SC substrate and $\mu_{SC} = 0$, $h_z = 4$, $|h_y| = 0.4$ and $|J| = 0.2$. This is the same case presented in the main article where we have considered a fixed singlet pairing field $\Delta = 1$ instead. Notice that the topological phase diagram is very similar to that presented in Fig. 2 of the main article. In Fig. S6 b) we present the lowest energy eigenstate for $\mu_{FM} = 4$ (left) and the corresponding Majorana modes localised at the edges of the wire. Moreover for the quasi-1D wire with width W=3 and length L=83 we solve Eq. S18 self-consistently for the pairing field Δ considering an attractive potential $U = 2$ only within the SC, while $|h_y| = 0.8$, $|J| = 0.2$, $\mu_{SC} = 0$ and $h_z = \mu_{FM} = 6$ for the FM wire (Fig. S6 c) and d)).

III. TOPOLOGICAL PHASE TRANSITIONS

In this section we review the topological criteria for 1D wires where SC fields and SOC are induced by proximity. For a translationally invariant system we consider only the local and nearest neighbors extended fields included in our discussion in previous sections. Introducing the spin-dependent Nambu spinor $\Psi_k = (c_{k,\uparrow}, c_{k,\downarrow}, c^{\dagger}_{-k,\uparrow}, c^{\dagger}_{-k,\downarrow})^T$ and the Pauli matrices $(\boldsymbol{\tau})$ for the particle-hole and $(\boldsymbol{\sigma})$ for the spin space, the Hamiltonian of the wire acquires in this case the following form

$$\mathcal{H} = \frac{1}{2}\sum_k \Psi^{\dagger}_k \left[(2t\cos k + \mu)\tau_3 - h\tau_3 \boldsymbol{d}_h \cdot \tilde{\boldsymbol{\sigma}} + \Delta\tau_2\sigma_2 + \sin k(\Delta^p \tau_1 i\sigma_2 \boldsymbol{d}_p + \Im\Delta^p \tau_2 i\sigma_2 \boldsymbol{d}_{\Im p} + \alpha \boldsymbol{d}_\alpha)\cdot\tilde{\boldsymbol{\sigma}}\right]\Psi_k\,, \quad \text{(S20)}$$

where $\tilde{\boldsymbol{\sigma}} = (\sigma_1, \tau_3\sigma_2, \sigma_3)$, h a Zeeman field with vector $\boldsymbol{d}_h$ in spin space, Δ a local SC field, μ and t the chemical potential and the nearest neighbors hopping term respectively, Δ^p and $\Im\Delta^p$ triplet nearest neighbors extended SC fields with a relative phase $\phi = \pi/2$ and a SO interactions. For the triplet fields we have considered the simplest odd in inversion function $\sin(k)$. The reality conditions in momentum space according to which free electron systems are classified to ten symmetry classes are $\Theta H(k)\Theta^{-1} = H(-k)$ and $\Xi H(k)\Xi^{-1} = -H(-k)$ where Θ and Ξ anti-unitary operators [7]. In the particular case with $\boldsymbol{d}_{\Im p} = \boldsymbol{d}_h \times \boldsymbol{d}_\alpha = \boldsymbol{d}_h \times \boldsymbol{d}_p$ and $\boldsymbol{d}_p = \boldsymbol{d}_\alpha$ the reality conditions are satisfied for $\Xi = \tau_1\mathcal{K}$ with $\Xi^2 = I$ and $\Theta = i\tau_3 \boldsymbol{d}_P \cdot i\sigma_2\tilde{\boldsymbol{\sigma}}\mathcal{K}$ with $\Theta^2 = I$. In addition a unitary chiral symmetry operator $\mathcal{S} = \Theta\cdot\Xi = \tau_2 \boldsymbol{d}_p \cdot i\sigma_2\tilde{\boldsymbol{\sigma}}$ anticommutes with the Hamiltonian. Thus when the triplet fields satisfy the condition $\boldsymbol{d}_{\Im p} = \boldsymbol{d}_h \times \boldsymbol{d}_\alpha = \boldsymbol{d}_h \times \boldsymbol{d}_p$, the system belongs to the BDI symmetry class and is characterised by a strong integer $\mathbb{Z}$ topological invariant.

Next we consider the particular case with $\boldsymbol{d}_h = (0,0,1)$, $\boldsymbol{d}_{\Im p} = (0,1,0)$ and $\boldsymbol{d}_\alpha = \boldsymbol{d}_p = (1,0,0)$ corresponding to the Hamiltonian Eq. S12 relevant to the case we study in the main article. Since in this case the system belongs to the BDI symmetry class with $\Theta = i\tau_3\sigma_3\mathcal{K}$ Eq. S20 acquires a block off-diagonal form $\mathcal{H}'(k) = \begin{pmatrix} 0 & \mathcal{A}(k) \\ \mathcal{A}(k)^{\dagger} & 0 \end{pmatrix}$ in the eigenbasis of the chiral operator $\mathcal{S} = \tau_2\sigma_3$. Therefore by applying the transformation $U_s = i[\tau_1 + i\tau_2 - \tau_3]\sigma_2 + [\tau_1 - i\tau_2 + \tau_3]\sigma_1$ we obtain $\mathcal{A}(k) = [-h^z + i\Im\Delta^p_y \sin k]\sigma_3 - [2t\cos k + \mu + i\Delta^p_x \sin k] + [a_x \sin k + i\Delta]\sigma_1$. We define as topological invariant the winding number [8]

$$\mathcal{W} = \frac{-i}{2\pi}\int_{k=0}^{k=2\pi} \frac{dz(k)}{z(k)} = \frac{1}{2\pi}\int_{k=0}^{k=2\pi} d\theta(k)\,, \quad \text{(S21)}$$

where $z(k)$ the unimodular complex number defined as $z(k) = exp[i\theta(k)] = Det(A(k))/|Det(A(k))| \in U(1)$. Since the one dimensional Hamiltonian Eq. S20, belongs to the BDI class, the topological charge $\mathcal{W}$ is in general an integer, $\mathcal{W} \in \mathbb{Z}$. In order to determine $\mathcal{W}$, for the particular system we get

$$\begin{aligned} z(k) &= \Re Det(A(k)) + i\Im Det(A(k)), \qquad \theta(k) = \tan^{-1}\left[\frac{\Im Det(A(k))}{\Re Det(A(k))}\right] \\ \Re Det(A(k)) &= \left[\Delta^2 + (2t\cos k + \mu)^2 - (h_z)^2 + ([\Im\Delta^p_y]^2 - \alpha_x^2 - [\Delta^p_x]^2)\sin^2 k\right] \\ \Im Det(A(k)) &= 2\left[\Delta\alpha_x - h_z\Im\Delta^p_y + (2tcosk + \mu)\Delta^p_x\right]\sin k \end{aligned} \quad \text{(S22)}$$

Topological phase transitions occur when the winding number is ill-defined which is the case when $|Det(A(k))| = 0$ or equivalently the Hamiltonian acquires zero eigenvalues. The quasiparticles eigenenergy spectrum of the particular Hamiltonian derives from the following equation

$$\begin{aligned} E(k) &= \pm\sqrt{h_z^2 + \Delta^2 + [2t\cos(k) + \mu]^2 + [[\Delta^p_x]^2 + a_x^2 + [\Im\Delta^p_y]^2]\sin^2(k) \pm 2\sqrt{B}} \\ B &= [\Delta\Delta^p_x + (2t\cos(k) + \mu)a_x]^2\sin^2(k) + [\Im\Delta^p_y\Delta^p_x\sin^2(k) - (2t\cos(k) + \mu)h_z]^2 + [\Im\Delta^p_y a_x \sin^2(k) + \Delta h_z]^2 \end{aligned} \quad \text{(S23)}$$

and for $k = 0, \pi$ acquires the following simple form

$$E_{s,m,\pm} = -sh_z + m\sqrt{\Delta^2 + [2t \pm \mu]^2} \quad \text{(S24)}$$

where $s = \pm$,$m = \pm$ and $E_{s,m,+}(E_{s,m,-})$ corresponds to $k = 0 (k = \pi)$.

In the following we derive the criteria with respect to the exchange energy h_z and the chemical potential μ of the wire, for topological charge $\mathcal{W} = 1$. From Eq. S24 we derive that for

$$|h_z| = \sqrt{(2t \pm \mu)^2 + \Delta^2}\,, \quad \text{(S25)}$$

the eigenenergy spectrum acquires a node at $k = 0(+)$ or $k = \pi$(-). For these momenta we get in general $\Im Det(A(0)) = \Im Det(A(\pi)) = 0$ and for

$$\begin{aligned} \sqrt{(2t-\mu)^2+\Delta^2} < |h_z| < \sqrt{(2t+\mu)^2+\Delta^2}, \quad \mu > 0 \\ \sqrt{(2t+\mu)^2+\Delta^2} < |h_z| < \sqrt{(2t-\mu)^2+\Delta^2}, \quad \mu < 0\,, \end{aligned} \tag{S26}$$

we observe that $\Re Det(A(0))\Re Det(A(\pi)) < 0$ and therefore $|\theta(0) - \theta(\pi)| = \pi$. Since $\theta(k)$ is a continuous function of momenta k and because $\theta(-k) = \theta(k) + \pi$ we conclude that for

$$\sqrt{(2t-|\mu|)^2+\Delta^2} < |h_z| < \sqrt{(2t+|\mu|)^2+\Delta^2} \tag{S27}$$

$\mathcal{W} = 1$. This is the condition for a topological non-trivial phase which can support a single pair of zero energy Majorana modes. Equivalently for the chemical potential we get the following condition

$$|2t - \sqrt{h_z^2 - \Delta^2}| < |\mu| < |2t + \sqrt{h_z^2 - \Delta^2}|\,. \tag{S28}$$

Finally we remark that when a charge current term J is present or the triplet fields don't satisfy the condition $\boldsymbol{d}_{\Im p} = \boldsymbol{d}_h \times \boldsymbol{d}_\alpha = \boldsymbol{d}_h \times \boldsymbol{d}_p$, as for example in the case of quasi-1D FM wire embedded in a SC where $\boldsymbol{d}_h = (1,0,1)$ and $\boldsymbol{d}_{\Im p} = \boldsymbol{d}_\alpha = \boldsymbol{d}_p = (1,1,0)$, reality condition $\Theta H(k)\Theta^{-1} = H(-k)$ cannot be satisfied for any anti-unitary operator. Therefore in this case the system breaks the chiral symmetry $\mathcal{S}$ and belongs to the D symmetry class with a strong $\mathbb{Z}_2$ topological invariant for one-dimensional systems.

[1] G. Varelogiannis, arXiv:1305.2976 (2013)

[2] G. Varelogiannis, Phys. Rev. Lett. **85**, 4172 (2000); A. Aperis, G. Varelogiannis, P.B. Littlewood and B.D. Simons, Journal of Physics Cond. Matter **20**, 434235 (2008); S. Tsonis, P. Kotetes, G. Varelogiannis, and P.B. Littlewood, ibid **20**, 434234 (2008); A. Aperis, G. Varelogiannis and P.B. Littlewood, Phys. Rev. Lett. **104**, 216403 (2010); P. Kotetes, A. Aperis and G. Varelogiannis, Philosophical Magazine **94**, 3789 (2014); G. Livanas, A. Aperis, P. Kotetes and G. Varelogiannis, Phys. Rev. B **91**, 104502 (2015).

[3] Y. Peng, F. Pientka, L. I. Glazman, and F. von Oppen Phys. Rev. Lett. **114**, 106801 (2015).

[4] M. Kjaergaard, K. Wolms, and K. Flensberg Phys. Rev. B **85**, 020503(R) (2012).

[5] A. C. Potter and P. A. Lee, Phys. Rev. Lett. 105, 227003 (2010).

[6] E. Dumitrescu, T.D. Stanescu, and S. Tewari Phys. Rev. B **91**, 121413(R)(2015).

[7] A. P. Schnyder, S. Ryu, A. Furusaki, and A.W.W. Ludwig, Phys. Rev. B **78**, 195125 (2008); IP Conf. Proc. 1134, **10** (2009).

[8] S. Tewari and J.D. Sau, Phys.Rev.Lett. **109**, 150408 (2012)
